# New Furan Derivatives from a Mangrove-Derived Endophytic Fungus *Coriolopsis* sp. J5

**DOI:** 10.3390/molecules22020261

**Published:** 2017-02-09

**Authors:** Liang-Liang Chen, Pei Wang, Hui-Qin Chen, Zhi-Kai Guo, Hao Wang, Hao-Fu Dai, Wen-Li Mei

**Affiliations:** Key Laboratory of Biology and Genetic Resources of Tropical Crops, Ministry of Agriculture, Institute of Tropical Bioscience and Biotechnology, Chinese Academy of Tropical Agricultural Sciences, Haikou 571101, China; chenliangliang502@163.com (L.-L.C.); wangpei@itbb.org.cn (P.W.); chenhuiqin@itbb.org.cn (H.-Q.C.); guozhikai@itbb.org.cn (Z.-K.G.); Hao.Wang@uni-duesseldorf.de (H.W.)

**Keywords:** *Coriolopsis* sp., *Ceriops tagal*, endophytic fungus, furan derivatives

## Abstract

Six new furan derivatives, named 5-(3-methoxy-3-oxopropyl)-furan-2-carboxylic acid (**1**), 1-(5-(2-hydroxypropanoyl)-furan-2-yl)-pentan-3-one (**2**), 2-hydroxy-1-(5-(1-hydroxypentyl)-furan-2-yl)-propan-1-one (**3**), 1-(5-(1,2-dihydroxypropyl)-furan-2-yl)-pentan-1-one (**4**), 5-(1-hydroxypent-4-en-1-yl)-furan-2-carboxylic acid (**5**) and 5-(3-hydroxypentyl)-furan-2-carboxylic acid (**6**), together with two new natural products, named 5-(1-hydroxypentyl)-furan-2-carboxylic acid (**7**) and (*E*)-5-(2-carboxyvinyl)-furan-2-carboxylic acid (**8**), were isolated from the solid rice fermentation of endophytic fungus *Coriolopsis* sp. J5, which was derived from mangrove plant *Ceriops tagal*. Their structures were unambiguously elucidated based on 1D and 2D NMR spectroscopy, and by HRESIMS measurements, as well as by comparison with the literature.

## 1. Introduction

Mangrove-derived endophytic fungi have played an important role in the discovery of new structures endowed with various bioactivities. Owing to the specific ecological circumstances of their hosts, related endophytes have to cope with both terrestrial and marine environments, resulting in a great microbial population diversity and metabolism specificity [1,2,3]. Furan derivatives are an important class of heterocyclic compounds that exhibit broad biological activities such as phytocidal, herbicidal and antioxidant, so that furan-containing molecules serve as privileged structures in pharmaceuticals [4,5]. However, furan derivatives from endophytic fungal cultures were reported rarely [6,7,8,9]. Previous chemical investigation on the mangrove endophytic fungus *Coriolopsis* sp. J5 has led to identify two furan derivatives, among which methyl 5-(2-methoxycarbonylethy)-furan-2-carboxylate showed inhibitory effect on *Xanthomonas axonopodis* [10], and the ongoing study on this fungus has now identified six new furan derivatives: 5-(3-methoxy-3-oxopropyl)-furan-2-carboxylic acid (**1**), 1-(5-(2-hydroxypropanoyl)-furan-2-yl)-pentan-3-one (**2**), 2-hydroxy-1-(5-(1-hydroxypentyl)-furan-2-yl)-propan-1-one (**3**), 1-(5-(1,2-dihydroxypropyl)-furan-2-yl)-pentan-1-one (**4**), 5-(1-hydroxy-pent-4-en-1-yl)-furan-2-carboxylic acid (**5**), 5-(3-hydroxypentyl)-furan-2-carboxylic acid (**6**), alongside two further derivatives 5-(1-hydroxypentyl)-furan-2-carboxylic acid (**7**) and (*E*)-5-(2-carboxyvinyl)-furan-2-carboxylic acid (**8**), herein first reported from a natural source. Hitherto, we report on the isolation and structural determination of these compounds.

## 2. Results and Discussion

**Figure 1:**
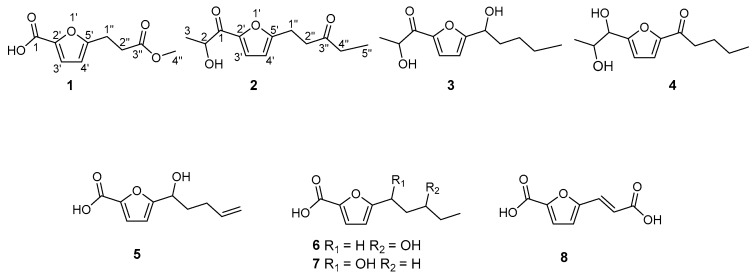
Structures of compounds **1**–**8**.

Compound **1** was obtained as a colorless oil. The molecular formula of **1** was deduced as C_9_H_10_O_5_ by the prominent ion peak at *m*/*z* 221.0421 [M + Na]^+^ (calcd. for C_9_H_10_O_5_Na, 221.0420) in the HRESIMS spectrum, corresponding to five degrees of unsaturation. This was corroborated by the ^13^C-NMR and DEPT data, which displayed nine resonances for four sp^2^ quaternary carbons (δ_C_ 172.7, 163.1, 160.0, 142.8), two sp^2^ methines (δ_C_ 121.3 and 108.8), two sp^3^ methylenes (δ_C_ 32.0 and 23.8) and one methoxyl group (δ_C_ 52.0). These observations, in combination with 2D NMR signals (the ^1^H-^1^H COSY cross-peak of H-3′/H-4′ and HMBC cross-peaks of H-3′/C-2′ and H-4′/C-5′), were consistent with the assumption that **1** possessed a ring and a carbonyl-conjugated diene (C-1, δ_C_ 160.0; C-2′, δ_C_ 142.8; C-3′, δ_C/H_ 121.3/7.10; C-4′, δ_C/H_ 108.8/6.21; C-5′, δ_C_ 163.1), indicating 2-furan-carboxylic acid unit in the structure [11]. The ^1^H-^1^H COSY correlation of H_2_-1′′/H_2_-2′′ and HMBC correlations from H_2_-1′′ (δ_H_ 3.04) to C-2′′ (δ_C_ 32.0) and C-3′′ (δ_C_ 172.7); from H_2_-2′′ (δ_H_ 2.71) to C-1′′ (δ_C_ 23.8) and C-3′′; as well as from H_3_-4′′ (3.68) to C-3′′ consisted of the methyl propionate moiety. Finally, the structure was constructed by attachment of the methyl propionate moiety to C-5′ through HMBC correlations from H_2_-1′′ to C-4′ and C-5′. On the basis of the above evidence, the structure of **1** was established as 5-(3-methoxy-3-oxopropyl)-furan-2-carboxylic acid (see Figure 1).

**Figure 2:**
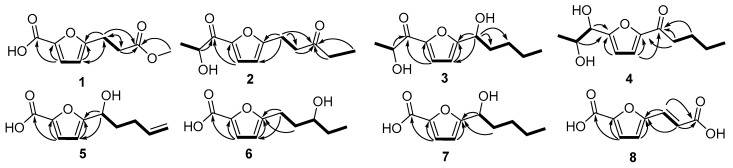
Key ^1^H-^1^H COSY (bold lines) and HMBC (arrows) correlations of compounds **1**–**8**.

By analysis of 1D and 2D NMR data (Table 1 and Table 2, Figure 2), compounds **2**–**8** were established as furan derivatives as well (see Supplementary Materials).

Compound **2** was obtained as a colorless oil. The molecular formula of **2** was deduced as C_12_H_16_O_4_ by HRESIMS prominent ion peak at *m*/*z* 225.1126 [M + H]^+^ (calcd. for C_12_H_17_O_4_, 225.1121) and ^13^C-NMR spectral data, indicating that the unsaturation degree of the molecule was five. By comparison of NMR data with those of **1**, the same furan core was inferred (C-2′, δ_C_ 148.3; C-3′, δ_C/H_ 121.0/7.21; C-4′, δ_C/H_ 109.2/6.24; C-5′, δ_C_ 161.3), and 2-hydroxypropanal unit attachment to C-2′ was deduced by ^1^H-^1^H COSY correlation of H-2 (δ_H_ 4.83)/H_3_-3(δ_H_ 1.46), as well as by HMBC correlations from H-2 to C-1 (δ_C_ 190.2), and from H-3′ to C-1 and C-2′. In addition, pentan-3-one located at C-5′ was implied by further ^1^H-^1^H COSY correlations of H_2_-1′′ (δ_H_ 3.01)/H_2_-2′′ (δ_H_ 2.83) and H_2_-4′′ (δ_H_ 2.44, 2.47)/H_3_-5′′ (δ_H_ 1.07), and combined with HMBC correlations from H_2_-2′′, H_2_-4′′ and H_3_-5′′ to C-3′′ (δ_C_ 209.1) and from H_2_-1′′ to C-4′, C-5′ and C-3′′. Based on the above evidence, compound **2** was determined as 1-(5-(2-hydroxypropanoyl)-furan-2-yl)-pentan-3-one (see Figure 1).

Compound **3** was obtained as a colorless oil. The molecular formula of **3** was deduced as C_12_H_18_O_4_ by HRESIMS prominent ion peak at *m*/*z* 249.1096 [M + Na]^+^ (calcd. for C_12_H_18_O_4_Na, 249.1097) and ^13^C-NMR spectral data, requiring four degrees of unsaturation in the structure, which was one degree reduced than that of **2**. By comparison, the ^1^H- and ^13^C-NMR data were similar to those of **2**, only differing in the presence of a hydroxylated methine (C-1′′, δ_C/H_ 69.9/4.75) instead of the carbonyl carbon at δ_C_ 209.1 (C-3′′) in **2**. Detailed analysis of the 1D and 2D NMR data, the moiety of pentan-1-ol was induced by ^1^H-^1^H COSY correlations of H-1′′ (δ_H_ 4.75)/H_2_-2′′ (δ_H_ 1.86)/H_2_-3′′ (δ_H_ 1.33)/H_2_-4′′ (δ_H_ 1.33)/H_3_-5′′ (δ_H_ 0.88). The pentan-1-ol moiety could be located at C-5′ based on HMBC correlations from H-1′′ to C-4′ (δ_C_ 108.7) and C-5′ (δ_C_ 163.2). Accordingly, compound **3** was determined as 2-hydroxy-1-(5-(1-hydroxy-pentyl)-furan-2-yl)-propan-1-one.

Compound **4** was obtained as a colorless oil. The molecular formula of **4** was deduced as C_12_H_18_O_4_ by HRESIMS prominent ion peak at *m*/*z* 249.1098 [M + Na]^+^ (calcd. for C_12_H_18_O_4_Na, 249.1097) and ^13^C-NMR spectral data. According to the NMR data, two aliphatic chains at C-2′ and C-5′ positions respectively of furan ring were constructed. The establishment of propane-1, 2-diol moiety and its attachment at C-2′ was achieved by ^1^H-^1^H COSY correlations of H-1 (δ_H_ 4.48)/H-2 (δ_H_ 4.01)/H_3_-3 (δ_H_ 1.12) and key HMBC correlations from H-1 and H-2 to C-2′ (δ_C_ 162.2), respectively. The chain at C-5′ was inferred to be pentan-1-one according to the ^1^H-^1^H COSY cross-peaks from H_2_-2′′ (δ_H_ 2.83) to H_3_-5′′ (0.95), and combined with the key HMBC correlations from H_2_-2′′ and H_2_-3′′ to C-1′′ (δ_C_ 191.7), and from H-4′ (δ_H_ 7.32) to C-1′′ and C-5′ (δ_C_ 153.0). Consideration of the above data led to the conclusion that **4** was 1-(5-(1,2-dihydroxypropyl)-furan-2-yl)-pentan-1-one.

Compound **5** was obtained as a colorless oil. The molecular formula of **5** was deduced as C_10_H_12_O_4_ by HRESIMS prominent ion peak at *m*/*z* 195.0661 [M − H]^−^ (calcd. for C_10_H_11_O_4_, 195.0663) and ^13^C-NMR spectral data. By comparison of the NMR data with those of **1**, the difference was at 5′-aliphatic chain. The pent-4-en-1-ol moiety at C-5′ was constructed based on the ^1^H-^1^H COSY correlations (Figure 2) of H-1′′ (δ_H_ 4.59)/H_2_-2′′ (δ_H_ 1.84)/H_2_-3′′ (δ_H_ 2.08)/H-4′′ (δ_H_ 5.73)/H_2_-5′′ (δ_H_ 4.95, 4.89) and the key HMBC correlations from H-1′′ to C-4′ (δ_C_ 107.7) and C-5′ (δ_C_ 161.4). Thus, the structure of **5** was supposed to be 5-(1-hydroxypent-4-en-1-yl)-furan-2-carboxylic acid.

Compound **6** was obtained as a colorless oil. The molecular formula of **6** was deduced as C_10_H_14_O_4_ by HRESIMS prominent ion peak at *m*/*z* 221.0785 [M + Na]^+^ (calcd. for C_10_H_14_O_4_Na, 221.0784). By comparison of the NMR data with those of **5**, both compounds possessed the same 2-furan-carboxylic acid skeleton. The remaining signals constructed of the pentan-3-ol moiety at C-5′, which was supported by ^1^H-^1^H COSY correlations of H_2_-1′′ (δ_H_ 2.91, 2.81)/H_2_-2′′ (δ_H_ 1.91, 1.77)/H-3′′ (δ_H_ 3.58)/H_2_-4′′ (δ_H_ 1.51)/H_3_-5′′ (δ_H_ 0.95), as well as the key HMBC correlations from H_2_-1′′ and H_2_-2′′ to C-5′ (δ_C_ 162.4). Thus, the structure of **6** was established as 5-(3-hydroxy-pentyl)-furan-2-carboxylic acid.

Compound **7** was obtained as a colorless oil. The ^1^H- and ^13^C-NMR data (Table 2) showed highly similarity to those of **6**. Detailed analysis of 2D NMR, the minor difference between these two compounds was the position of the hydroxyl group at 5′-aliphatic chain. Instead of 3′′-OH in **6**, **7** possessed 1′′-OH, which was induced by ^1^H-^1^H COSY correlations of H-1′′ (δ_H_ 4.68)/H_2_-2′′ (δ_H_ 1.81)/H_2_-3′′ (δ_H_ 1.23)/H_2_-4′′ (δ_H_ 1.23)/H_3_-5′′ (δ_H_ 0.79) and the key HMBC correlations from H-1′′ and H_2_-2′′ to C-5′ (δ_C_ 162.9). Hence, compound **7** was determined as 5-(1-hydroxypentyl)-furan-2-carboxylic acid, which was corresponding with the molecular weight observed in ESIMS spectrum with the positive ion peak at *m*/*z* 221.1 [M + Na]^+^.

Compound **8** was isolated as a pale yellow powder. The ^1^H- and ^13^C-NMR data (Table 2) established a 2-furancarboxylic acid unit in the structure. Similarly, the remaining signals consisted of acrylic acid by the ^1^H-^1^H COSY cross-peak of H-1′′ (δ_H_ 7.41)/H-2′′ (δ_H_ 6.34) and key HMBC correlations from H-1′′ and H-2′′ to C-3′′ (δ_C_ 167.1). The attachment to C-5′ was inferred by HMBC correlations from H-1′′ and H-2′′ to C-5′ (δ_C_ 153.2). The large ^3^*J* coupling constant between H-1′′ and H-2′′ (15.9 Hz) suggested the geometry of the double bond at C-1′′/C-2′′ to be *E* [12]. Thus, compound **8** was determined as (*E*)-5-(2-carboxyvinyl)-furan-2-carboxylic acid, which was corresponding with the molecular weight observed in ESIMS spectrum with the positive ion peak at *m*/*z* 205.1 [M + Na]^+^. To the best of our knowledge, compounds **7** and **8** are commercially available, however, this was the first report of their isolation from Nature. Assessment of the absolute configurations of compounds **2**–**7** could not be performed due to the minute amounts of metabolites remaining after bioactivity assays.

Since an even number of carbon atoms was observed in the structural skeletons of the isolated substances, these furans most likely arise through the polyketide pathway [7]. 

All the compounds were evaluated for their cytotoxic activities against three human cell lines (K562, SGC-7901 and BEL-7402) and antibacterial activities against *Staphylococcus aureus* (ATCC51650), *Ralstonia solanacearum*, *Fusarium oxysporum* f. sp. *cubense* race 4, *Fusarium oxysporum* f. sp. *niveum*, *Fusarium oxysporum* f. sp. *vasinfectum* and *Candida albicans* (ATCC10231). However, none of these compounds showed any obvious cytotoxic or antibacterial activities.

## 3. Experimental Section

### 3.1. General

The NMR spectra were recorded on an AV-500 spectrometer (500 MHz for ^1^H-NMR and 125 MHz for ^13^C-NMR; Bruker, Billerica, MA, USA), using the solvent residue signal as the internal standard. Optical rotations were recorded using a Rudolph Autopol III polarimeter (Rudolph Research Analytical, Hackettstown, NJ, USA). The UV spectra were obtained from a DU-800 spectrometer (Beckman, Brea, CA, USA). The IR spectra (KBr pellets) were run on a 380 FT-IR instrument from Nicolet (Thermo, Pittsburgh, PA, USA). Column chromatography was performed with ODS gel (20–45 mm, Fuji Silysia Chemical Co. Ltd., Durham, NC, USA), Sephadex LH-20 (Merck, Darmstadt, Germany) and silica gel (60–80, 200–300 mesh, Qingdao Haiyang Chemical Co. Ltd., Qingdao, China). TLC analyses were carried out on silica gel G precoated plates (Qingdao Haiyang Chemical Co. Ltd.), and spots were detected by spraying with 5% H_2_SO_4_ in EtOH followed by heating. 

### 3.2. Fungal Material

The mangrove endophytic fungus *Coriolopsis* sp. J5 used in this study was isolated from healthy branches of *Ceriops tagal* (*Rhizophoraceae*), which were collected in July 2011 from Dong Zhai Gang Mangrove National Nature Reserve in Hainan Island, China. The fungus was identified by sequence analysis of the ITS region of its 18 s rDNA. A BLAST search result indicated that the sequence was most similar (99%) to the sequence of *Coriolopsis* sp. (compared to AY336771.1). The reserved sample was stored at the Institute of Tropical Bioscience and Biotechnology, Chinese Academy of Tropical Agricultural Sciences (Haikou, China), and maintained on potato dextrose agar (PDA) slant at 4 °C.

### 3.3. Fermentation, Extraction and Isolation

The fungus stored on PDA medium was inoculated and cultured on PDA agar for 7 days. Seed medium (potato 200 g, dextrose 20 g, distilled water 1000 mL) in 500 mL × 10 Erlenmeyer flasks was inoculated with fungus and incubated at room temperature for 4 days on a rotating shaker (120 rpm). Production medium of solid rice in 1000 mL flasks (rice 80 g, distilled water 120 mL for each) was inoculated with seed solution 10 mL for each. Flask cultures were incubated at room temperature under static conditions and daylight for 60 days, and cultures from 200 flasks were harvested for the isolation of substances. Each fungal culture was diced and extracted with EtOH, followed by filter through cheesecloth. Subsequently, the filtrate was extracted three times with an equal volume of Petroleum ether, EtOAc and *n*-BuOH, successively. 

The EtOAc fraction (129.0 g) was chromatographed on a silica gel column using a step gradient elution of CHCl_3_–MeOH (1:0–0:1, *v*/*v*) to afford ten fractions (Fr.1–Fr.10). Fr.3 (1.5 g) was applied to octadecyl silane (ODS) gel column with gradient elution of MeOH–H_2_O (3:7, 4:6, 5:5, 6:4, 7:3, 8:2, 9:1, 1:0) to yield 19 subfractions (Fr.A1–Fr.A19). Fr.A3 (39.3 mg) was chromatographed on a Sephadex LH-20 column eluting with MeOH and then submitted to silica gel column with gradient elution of petroleum ether (PE)–EtOAc (10:1) to give compound **2** (4.5 mg). Fr.5 (9.9 g) was applied to ODS column with gradient elution of MeOH–H_2_O (3:7, 4:6, 5:5, 6:4, 7:3, 8:2, 9:1, 1:0) to yield 19 subfractions (Fr.B1–Fr.B19). Subfraction Fr.B5 (607.1 mg) was chromatographed on a Sephadex LH-20 column eluting with CHCl_3_/MeOH (1:1) and then submitted to silica gel column with gradient elution of PE–EtOAc (20:1, 18:1, 15:1, 12:1) to give compounds **1** (86.0 mg) and **3** (14.6 mg). Fr.6 (4.2 g) was chromatographed on a Sephadex LH-20 column eluting with CHCl_3_/MeOH (1:1) to produce 7 subfractions (Fr.C1–Fr.C7). Subfraction Fr.C6 was chromatographed on a Sephadex LH-20 column eluting with MeOH, then followed by silica gel column chromatography and eluted with PE/acetone (8:1) to get compound **4** (10.0 mg). Fr.7 (20.0 g) was applied to ODS column with gradient elution of MeOH–H_2_O (3:7, 4:6, 5:5, 6:4, 7:3, 8:2, 9:1, 1:0) to yield 12 subfractions (Fr.D1–Fr.D12). Subfraction Fr.D3 was chromatographed on a Sephadex LH-20 column eluting with MeOH and then separated on silica gel column with PE/acetone (9:1, 7:1) as eluent, followed by purified on Sephadex LH-20 column eluting with acetone to give compounds **5** (2.5 mg) and **6** (10.2 mg). Subfraction Fr.D4 was chromatographed on a Sephadex LH-20 column eluting with MeOH and then submitted to silica gel column with PE/acetone (7:1) as eluent, and further purified by Sephadex LH-20 column eluting with acetone to obtain compound **7** (46.5 mg). Subfraction Fr.D1 was chromatographed on a Sephadex LH-20 column eluting with MeOH to get compound **8** (27.4 mg).

*5-(2-Methoxycarbonyl-ethyl)-furan-2-carboxylic acid* (**1**): colorless oil; UV (MeOH) λ_max_ (log ε) 240 (0.46), 264 (0.45) nm; ^1^H- and ^13^C-NMR data: Table 1 and Table 2; HRESIMS *m*/*z* 221.0421 [M + Na]^+^ (calcd. for C_9_H_10_O_5_Na, 221.0420, error −0.8 ppm).

*1-(5-(1,2-Dihydroxy-propyl)-furan-2-yl)-pentan-1-one* (**2**): colorless oil; ${\left[\alpha \right]}_{D}^{25}$ +3.0 (*c* 0.05, CHCl_3_); UV (MeOH) λ_max_ (log ε) 240 (0.22), 288 (0.63) nm; ^1^H- and ^13^C-NMR data: Table 1 and Table 2; HRESIMS *m*/*z* 225.1126 [M + H]^+^ (calcd. for C_12_H_17_O_4_, 225.1121, error −2.2 ppm).

*2-Hydroxy-1-(5-(1-hydroxy-pentyl)-furan-2-yl)propan-1-one* (**3**): colorless oil; ${\left[\alpha \right]}_{D}^{25}$ −3.3 (c 0.05, CHCl_3_); UV (MeOH) λ_max_ (log ε) 240 (0.35), 284 (0.94) nm; ^1^H- and ^13^C-NMR data: Table 1 and Table 2; HRESIMS *m*/*z* 249.1096 [M + Na]^+^ (calcd. for C_12_H_18_O_4_Na, 249.1097, error −0.4 ppm).

*5-(1-Hydroxy-pent-4-enyl)-furan-2-carboxylic acid* (**4**): colorless oil; ${\left[\alpha \right]}_{D}^{25}$ +7.9 (c 0.05, CHCl_3_); UV (MeOH) λ_max_ (log ε) 240 (0.20), 276 (0.57) nm; ^1^H- and ^13^C-NMR data: Table 1 and Table 2; HRESIMS *m*/*z* 249.1098 [M + Na]^+^ (calcd. for C_12_H_18_O_4_Na, 249.1097, error −0.3 ppm).

*5-(3-Hydroxy-pentyl)-furan-2-carboxylic acid* (**5**): colorless oil; ${\left[\alpha \right]}_{D}^{25}$ +2.0 (c 0.05, CHCl_3_); UV (MeOH) λ_max_ (log ε) 240 (0.23), 260 (0.32) nm; ^1^H- and ^13^C-NMR data: Table 2 and Table 3; HRESIMS *m*/*z* 195.0661 [M − H]^−^ (calcd. for C_10_H_11_O_4_, 195.0663, error −0.1 ppm).

*5-(1-Hydroxy-pentyl)-furan-2-carboxylic acid* (**6**): colorless oil; ${\left[\alpha \right]}_{D}^{25}$ +2.4 (c 0.05, CHCl_3_); UV (MeOH) λ_max_ (log ε) 240 (0.20), 266 (0.24) nm; ^1^H- and ^13^C-NMR data: Table 2 and Table 3; HRESIMS *m*/*z* 221.0785 [M + Na]^+^ (calcd. for C_10_H_14_O_4_Na, 221.0784, error 0 ppm).

### 3.4. Biological Assays

Cytotoxic activity was tested by the MTT method as described previously [13]. Three cancer cell lines, human chronic myelogenous leukemia cell line (K562), human gastric carcinoma cell line (SGC-7901), and human hepatocellular carcinoma (BEL-7402) were used. Antibacterial activity was determined by the filter paper disc diffusion method [14] against *Staphylococcus aureus* (ATCC51650), *Ralstonia solanacearum*, *Fusarium oxysporum* f. sp. *cubense* race 4, *Fusarium oxysporum* f. sp. *niveum*, *Fusarium oxysporum* f. sp. *vasinfectum* and *Candida albicans* (ATCC10231).

## Figures and Tables

**Table 1:** ^1^H-NMR data for **1**–**4** (**1**, **2** and **3** in CDCl_3_, **4** in CD_3_OD, 500 MHz, *J* in Hz, δ in ppm).

No.	1	2	3	4
1				4.48 (1H, d, *J* = 6.0)
2	-	4.83 (1H, q, *J* = 6.9)	4.86 (1H, q, *J* = 7.0)	4.01 (1H, dq, *J* = 6.4, 6.0)
3	-	1.46 (3H, d, *J* = 6.9)	1.46 (3H, d, *J* = 7.0)	1.12 (3H, d, *J* = 6.4)
3′	7.10 (1H, d, *J* = 3.4)	7.21 (1H, d, *J* = 3.4)	7.24 (1H, d, *J* = 3.5)	6.54 d (1H, d, *J* = 3.6)
4′	6.21 d (1H, d, *J* = 3.4)	6.24 (1H, d, *J* = 3.4)	6.43 (1H, d, *J* = 3.5)	7.32 d (1H, d, *J* = 3.6)
1′′	3.04 (2H, dd, *J* = 7.5)	3.01 (2H, dd, *J* = 7.2)	4.75 (1H, m)	
2′′	2.71 (2H, dd, *J* = 7.5)	2.83 (2H, dd, *J* = 7.2)	1.86 (2H, m)	2.83 (2H, dd, *J* = 7.5, 7.4)
3′′	-	-	1.33 (2H, m)	1.66 (2H, m)
4′′	3.68 (3H, s)	2.44 (1H, dd, *J* = 7.3)	1.33 (2H, m)	1.39 (2H, m)
		2.47 (1H, dd, *J* = 7.3)		
5′′	-	1.07 (3H, t, *J* = 7.3)	0.88 (3H, d, *J* = 7.2)	0.95 (3H, t, *J* = 7.4)

**Table 2:** ^13^C-NMRdata for **1**–**8** (**1**, **2**, **3**, **5** and **6** in CDCl_3_, **4** and **7** in CD_3_OD, **8** in DMSO-*d*_6_, 125 MHz, *J* in Hz, δ in ppm).

No.	1	2	3	4	5	6	7	8
1	160.0	190.2	190.2	73.4	161.6	163.1	163.2	159.3
2		69.5	69.7	70.6				
3		22.4	22.1	19.1				
2′	142.8	148.3	149.0	162.2	145.0	142.4	146.4	146.2
3′	121.3	121.0	120.5	110.9	118.6	121.5	119.0	119.6
4′	108.8	109.2	108.7	120.3	107.7	108.4	108.6	116.3
5′	163.1	161.3	163.2	153.0	161.4	162.4	162.9	153.2
1′′	23.8	22.5	69.9	191.7	66.6	24.9	68.2	130.4
2′′	32.0	39.7	35.5	37.2	34.5	34.8	36.5	119.9
3′′	172.7	209.1	27.5	26.5	29.5	72.6	28.6	167.1
4′′	52.0	36.1	22.5	23.4	137.6	30.4	23.4	
5′′		7.9	14.1	14.2	115.1	10.0	14.4	

**Table 3:** ^1^H-NMR data for **5**–**8** (**5** and **6** in CDCl_3_, **7** in CD_3_OD, **8** in DMSO-*d*_6_, 500 MHz, *J* in Hz, δ in ppm).

No.	5	6	7	8
3′	7.02 (1H, d, *J* = 3.5)	7.23 (1H, d, *J* = 3.4)	7.16 (1H, d, *J* =3.4)	7.26 (1H, d, *J* =3.5)
4′	6.26 (1H, d, *J* = 3.5)	6.20 (1H, d, *J* = 3.4)	6.43 (1H, d, *J* =3.4)	7.03 (1H, d, *J* =3.5)
1′′	4.59 (1H, m)	2.91 (1H, m)	4.68 (1H, m)	7.41 (1H, d, *J* = 15.9)
		2.81 (1H, m)		
2′′	1.84 (2H, m)	1.91 (1H, tdd, *J* = 3.7, 6.9, 13.4)	1.81 (2H, m)	6.34 (1H, d, *J* = 15.9)
		1.77 (1H, m)		
3′′	2.08 (2H, m)	3.58 (1H, m)	1.23 (2H, m)	-
4′′	5.73 (1H, m)	1.51 (2H, m)	1.23 (2H, m)	-
5′′	4.95 (1H, d, *J* = 17.2) 4.89 (1H, d, *J* = 10.0)	0.95 (3H, t, *J* = 7.5)	0.79 (3H, t, *J* = 7.2)	-

## Supplementary Materials

The following are available online at: http://www.mdpi.com/1420-3049/22/2/261/s1. The original spectra of NMR and HRESIMS or ESIMS data for the compounds **1**–**6** are available as Supplementary Materials.
