# Supplementary material for: New Furan Derivatives from a Mangrove-Derived Endophytic Fungus Coriolopsis sp. J5
Source: Molecules. 2017 Feb 9;22(2):261. doi: 10.3390/molecules22020261 (PMC6155590; doi:10.3390/molecules22020261)
Supplement: Supplementary file 1 [file molecules-22-00261-s001.pdf]

# Supplementary Materials: New Furan Derivatives from a Mangrove-Derived Endophytic Fungus *Coriolopsis* sp. J5

**Liang-Liang Chen, Pei Wang, Hui-Qin Chen, Zhi-Kai Guo, Hao Wang, Hao-Fu Dai and Wen-Li Mei**

## Table of Contents

**Figure S1.** HRESIMS spectrum of 5-(3-methoxy-3-oxopropyl)-furan-2-carboxylic acid (**1**)
**Figure S2.** $^{1}$H-NMR (500 MHz, $CDCl_3$) spectrum of 5-(3-methoxy-3-oxopropyl)-furan-2-carboxylic acid (**1**)
**Figure S3.** $^{13}$C-NMR (125 MHz, $CDCl_3$) spectrum of 5-(3-methoxy-3-oxopropyl)-furan-2-carboxylic acid (**1**)
**Figure S4.** $^{1}$H-$^{1}$H COSY (500 MHz, $CDCl_3$) spectrum of 5-(3-methoxy-3-oxopropyl)-furan-2-carboxylic acid (**1**)
**Figure S5.** HSQC (500 MHz, $CDCl_3$) spectrum of 5-(3-methoxy-3-oxopropyl)-furan-2-carboxylic acid (**1**)
**Figure S6.** HMBC (500 MHz, $CDCl_3$) spectrum of 5-(3-methoxy-3-oxopropyl)-furan-2-carboxylic acid (**1**)
**Figure S7.** HRESIMS spectrum of 1-(5-(2-hydroxypropanoyl)-furan-2-yl)-pentan-3-one (**2**)
**Figure S8.** $^{1}$H-NMR (500 MHz, $CDCl_3$) spectrum of 1-(5-(2-hydroxypropanoyl)-furan-2-yl)-pentan-3-one (**2**)
**Figure S9.** $^{13}$C-NMR (125 MHz, $CDCl_3$) spectrum of 1-(5-(2-hydroxypropanoyl)-furan-2-yl)-pentan-3-one (**2**)
**Figure S10.** $^{1}$H-$^{1}$H COSY (125 MHz, $CDCl_3$) spectrum of 1-(5-(2-hydroxypropanoyl)-furan-2-yl)-pentan-3-one (**2**)
**Figure S11.** HSQC (125 MHz, $CDCl_3$) spectrum of 1-(5-(2-hydroxypropanoyl)-furan-2-yl)-pentan-3-one (**2**)
**Figure S12.** HMBC (125 MHz, $CDCl_3$) spectrum of 1-(5-(2-hydroxypropanoyl)-furan-2-yl)-pentan-3-one (**2**)
**Figure S13.** HRESIMS spectrum of 2-hydroxy-1-(5-(1-hydroxypentyl)-furan-2-yl)-propan-1-one (**3**)
**Figure S14.** $^{1}$H-NMR (500 MHz, $CDCl_3$) spectrum of 2-hydroxy-1-(5-(1-hydroxypentyl)-furan-2-yl)-propan-1-one (**3**)
**Figure S15.** $^{13}$C-NMR (125 MHz, $CDCl_3$) spectrum of 2-hydroxy-1-(5-(1-hydroxypentyl)-furan-2-yl)-propan-1-one (**3**)
**Figure S16.** $^{1}$H-$^{1}$H COSY (500 MHz, $CDCl_3$) spectrum of 2-hydroxy-1-(5-(1-hydroxypentyl)-furan-2-yl)-propan-1-one (**3**)
**Figure S17.** HSQC (500 MHz, $CDCl_3$) spectrum of 2-hydroxy-1-(5-(1-hydroxypentyl)-furan-2-yl)-propan-1-one (**3**)
**Figure S18.** HMBC (500 MHz, $CDCl_3$) spectrum of 2-hydroxy-1-(5-(1-hydroxypentyl)-furan-2-yl)-propan-1-one (**3**)
**Figure S19.** HRESIMS spectrum of 1-(5-(1,2-dihydroxypropyl)-furan-2-yl)-pentan-1-one (**4**)
**Figure S20.** $^{1}$H-NMR (500 MHz, $CDCl_3$) spectrum of 1-(5-(1,2-dihydroxypropyl)-furan-2-yl)-pentan-1-one (**4**)
**Figure S21.** $^{13}$C-NMR (125 MHz, $CDCl_3$) spectrum of 1-(5-(1,2-dihydroxypropyl)-furan-2-yl)-pentan-1-one (**4**)
**Figure S22.** $^{1}$H-$^{1}$H COSY (500 MHz, $CDCl_3$) spectrum of 1-(5-(1,2-dihydroxypropyl)-furan-2-yl)-pentan-1-one (**4**)
**Figure S23.** HSQC (500 MHz, $CDCl_3$) spectrum of 1-(5-(1,2-dihydroxypropyl)-furan-2-yl)-pentan-1-one (**4**)
**Figure S24.** HMBC (500 MHz, $CDCl_3$) spectrum of 1-(5-(1,2-dihydroxypropyl)-furan-2-yl)-pentan-1-one (**4**)
**Figure S25.** HRESIMS spectrum of 5-(1-hydroxypent-4-en-1-yl)-furan-2-carboxylic acid (**5**)
**Figure S26.** $^{1}$H-NMR (500 MHz, $CDCl_3$) spectrum of 5-(1-hydroxypent-4-en-1-yl)-furan-2-carboxylic acid (**5**)
**Figure S27.** $^{13}$C-NMR (125 MHz, $CDCl_3$) spectrum of 5-(1-hydroxypent-4-en-1-yl)-furan-2-carboxylic acid (**5**)
**Figure S28.** $^{1}$H-$^{1}$H COSY (500 MHz, $CDCl_3$) spectrum of 5-(1-hydroxypent-4-en-1-yl)-furan-2-carboxylic acid (**5**)
**Figure S29.** HSQC (500 MHz, $CDCl_3$) spectrum of 5-(1-hydroxypent-4-en-1-yl)-furan-2-carboxylic acid (**5**)
**Figure S30.** HMBC (500 MHz, $CDCl_3$) spectrum of 5-(1-hydroxypent-4-en-1-yl)-furan-2-carboxylic acid (**5**)
**Figure S31.** HRESIMS spectrum of 5-(3-hydroxypentyl)-furan-2-carboxylic acid (**6**)
**Figure S32.** $^{1}$H-NMR (500 MHz, $CDCl_3$) spectrum of 5-(3-hydroxypentyl)-furan-2-carboxylic acid (**6**)
**Figure S33.** $^{13}$C-NMR (125 MHz, $CDCl_3$) spectrum of 5-(3-hydroxypentyl)-furan-2-carboxylic acid (**6**)
**Figure S34.** $^{1}$H-$^{1}$H COSY (500 MHz, $CDCl_3$) spectrum of 5-(3-hydroxypentyl)-furan-2-carboxylic acid (**6**)
**Figure S35.** HSQC (500 MHz, $CDCl_3$) spectrum of 5-(3-hydroxypentyl)-furan-2-carboxylic acid (**6**)
**Figure S36.** HMBC (500 MHz, $CDCl_3$) spectrum of 5-(3-hydroxypentyl)-furan-2-carboxylic acid (**6**)

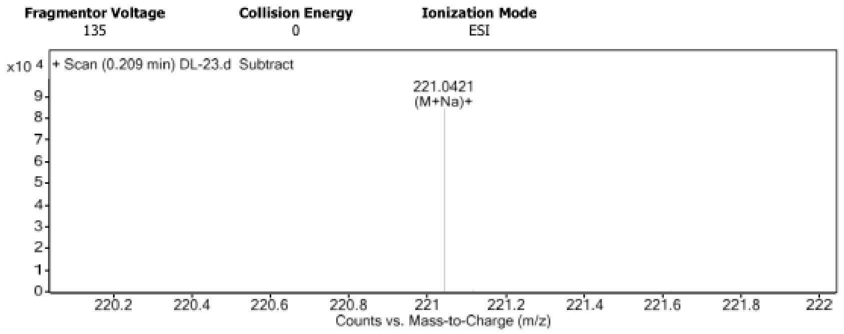


| Formula | CalculatedMass | CalculatedMz | Mz | Diff. (mDa) | Diff. (ppm) | DBE |
|---|---|---|---|---|---|---|
| C9 H10 O5 | 198.0528 | 221.0420 | 221.0421 | -0.2 | -0.8 | 5.0000 |

**Figure S1.** HRESIMS spectrum of 5-(3-methoxy-3-oxopropyl)-furan-2-carboxylic acid (**1**).

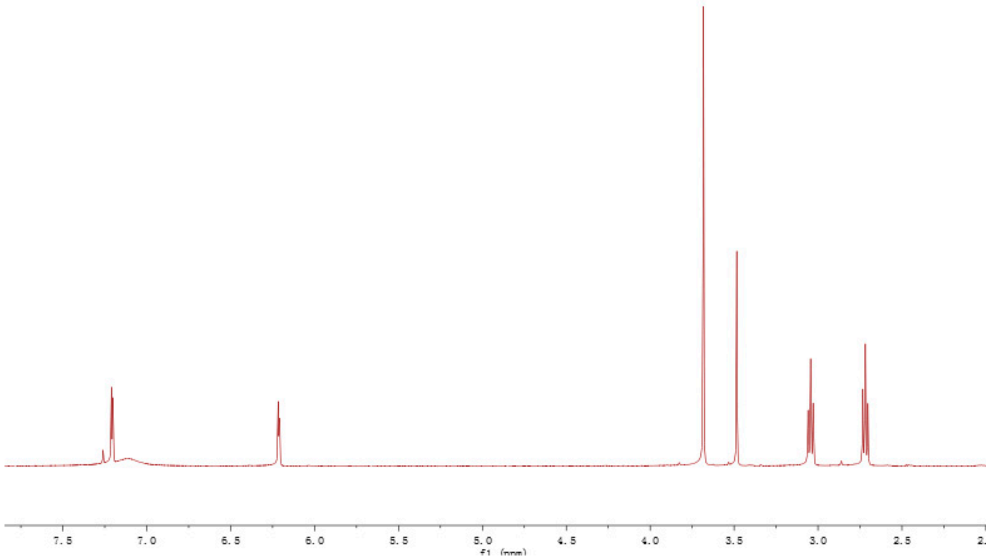


**Figure S2.** $^{1}$H-NMR (500 MHz, $CDCl_3$) spectrum of 5-(3-methoxy-3-oxopropyl)-furan-2-carboxylic acid (**1**).

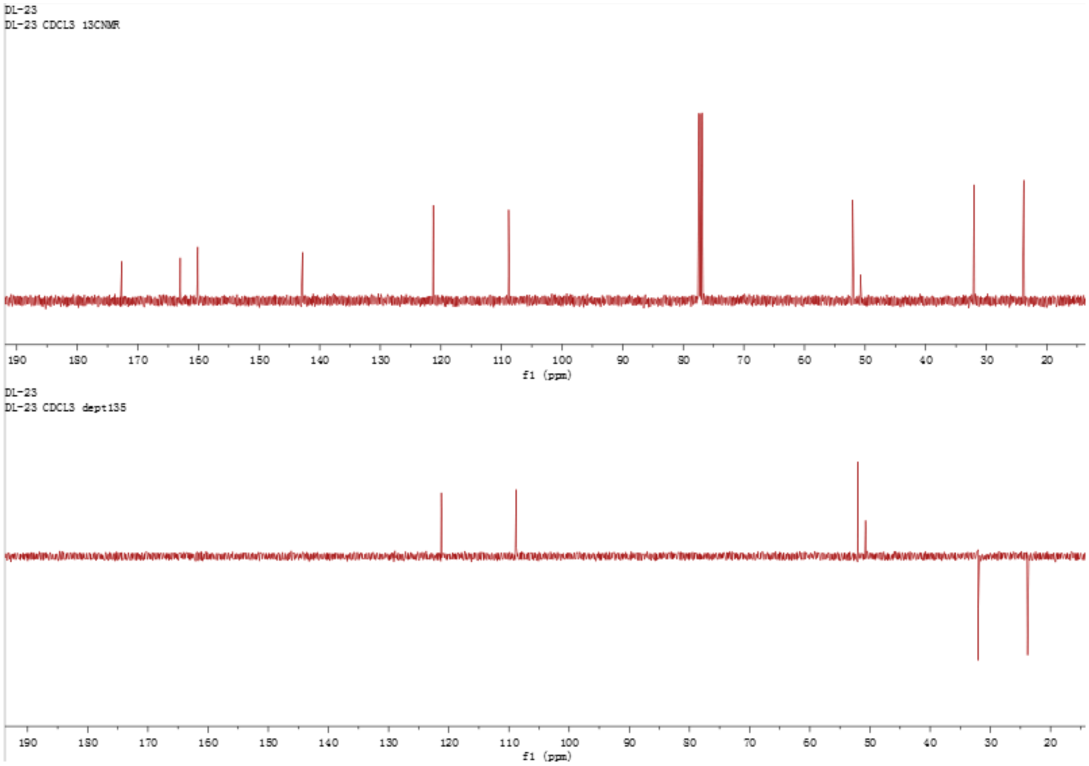


**Figure S3.** $^{13}$C-NMR (125 MHz, $CDCl_3$) spectrum of 5-(3-methoxy-3-oxopropyl)-furan-2-carboxylic acid (**1**).

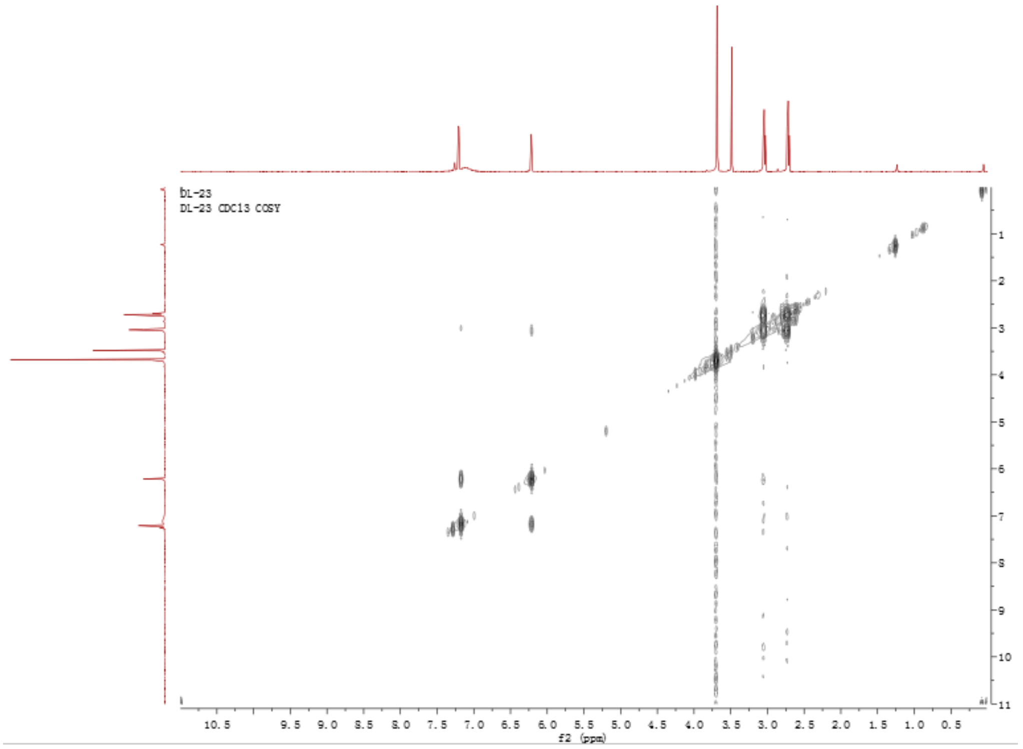


**Figure S4.** $^{1}$H-$^{1}$H COSY (500 MHz, $CDCl_3$) spectrum of 5-(3-methoxy-3-oxopropyl)-furan-2-carboxylic acid (**1**).

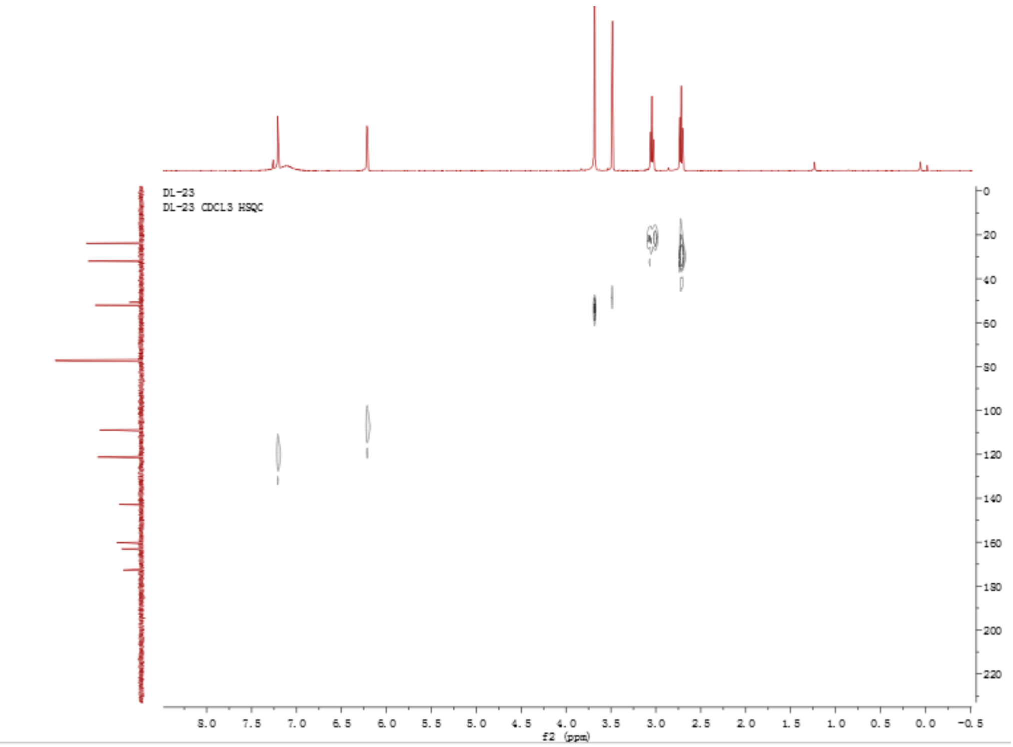


**Figure S5.** HSQC (500 MHz, $CDCl_3$) spectrum of 5-(3-methoxy-3-oxopropyl)-furan-2-carboxylic acid (**1**).

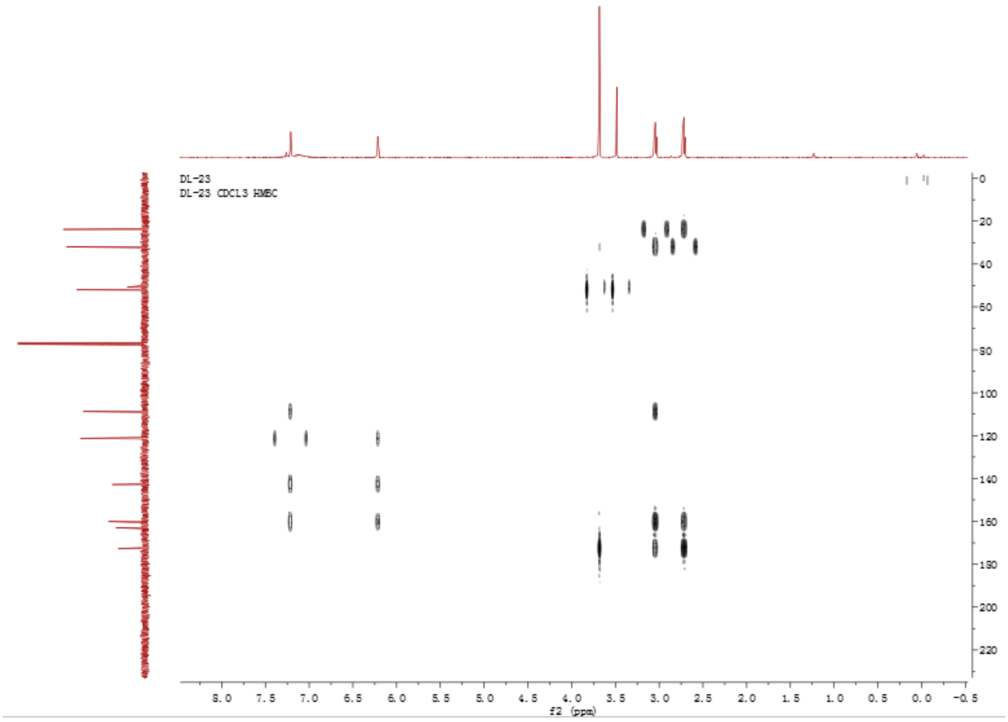


**Figure S6.** HMBC (500 MHz, $CDCl_3$) spectrum of 5-(3-methoxy-3-oxopropyl)-furan-2-carboxylic acid (**1**).

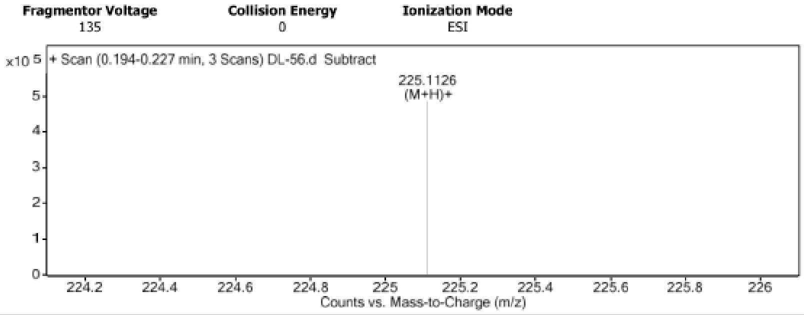


| Formula | CalculatedMass | CalculatedMz | Mz | Diff. (mDa) | Diff. (ppm) | DBE |
|---|---|---|---|---|---|---|
| C12 H16 O4 | 224.1049 | 225.1121 | 225.1126 | -0.5 | -2.2 | 5.0000 |

**Figure S7.** HRESIMS spectrum of 1-(5-(2-hydroxypropanoyl)-furan-2-yl)-pentan-3-one (**2**).

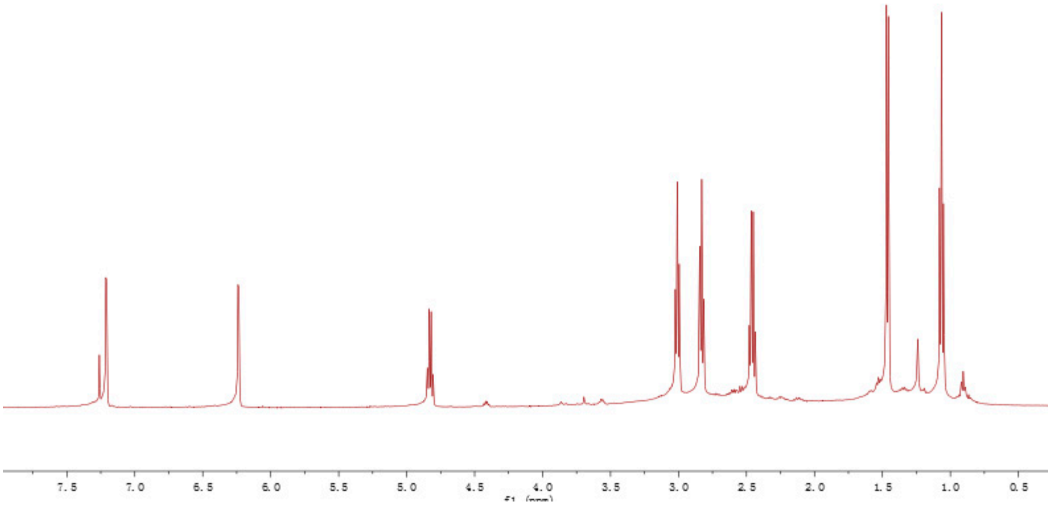

**Figure S8.** $^{1}$H-NMR (500 MHz, $CDCl_3$) spectrum of 1-(5-(2-hydroxypropanoyl)-furan-2-yl)-pentan-3-one (**2**).

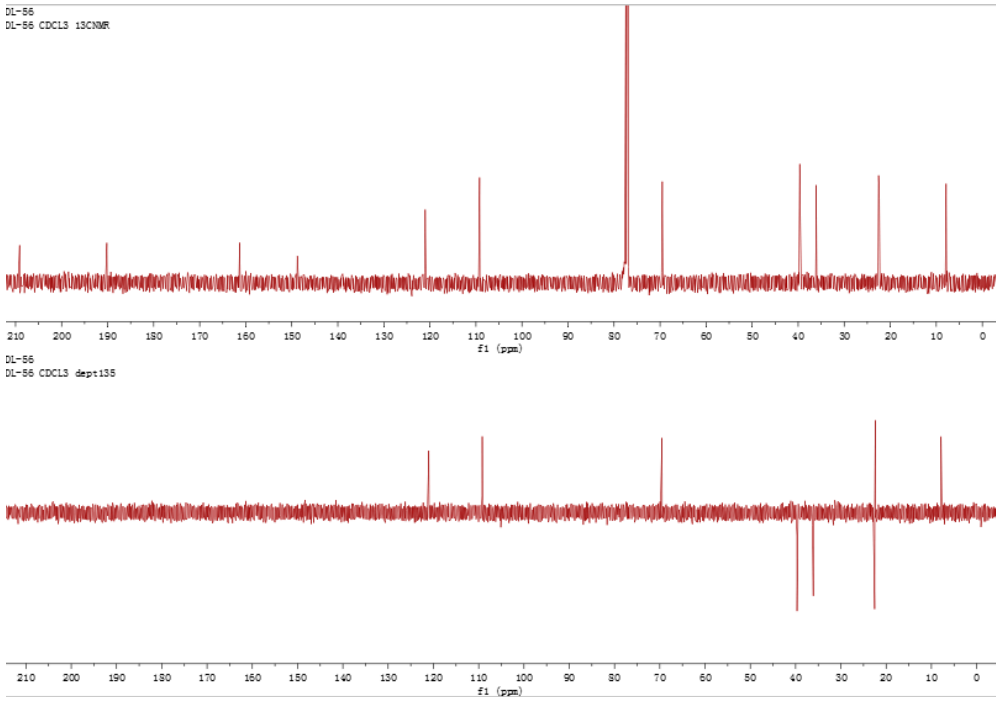


**Figure S9.** $^{13}$C-NMR (125 MHz, $CDCl_3$) spectrum of 1-(5-(2-hydroxypropanoyl)-furan-2-yl)-pentan-3-one (**2**).

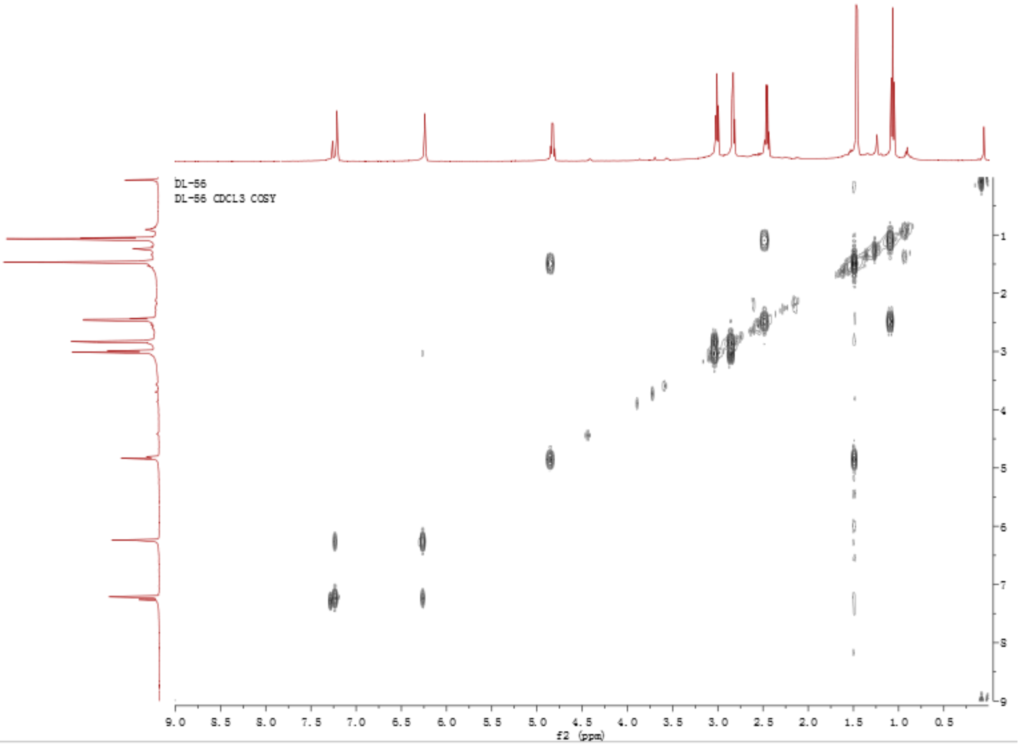


**Figure S10.** $^{1}$H-$^{1}$H COSY (500 MHz, $CDCl_3$) spectrum of 1-(5-(2-hydroxypropanoyl)-furan-2-yl)-pentan-3-one (**2**).

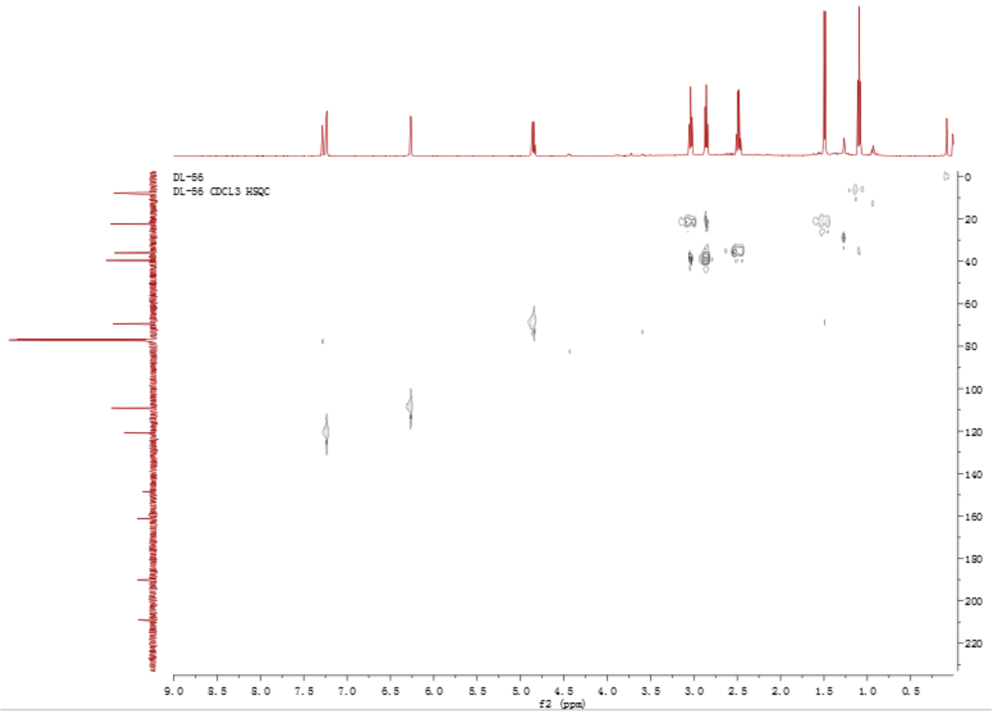


**Figure S11.** HSQC (500 MHz, $CDCl_3$) spectrum of 1-(5-(2-hydroxypropanoyl)-furan-2-yl)-pentan-3-one (**2**).

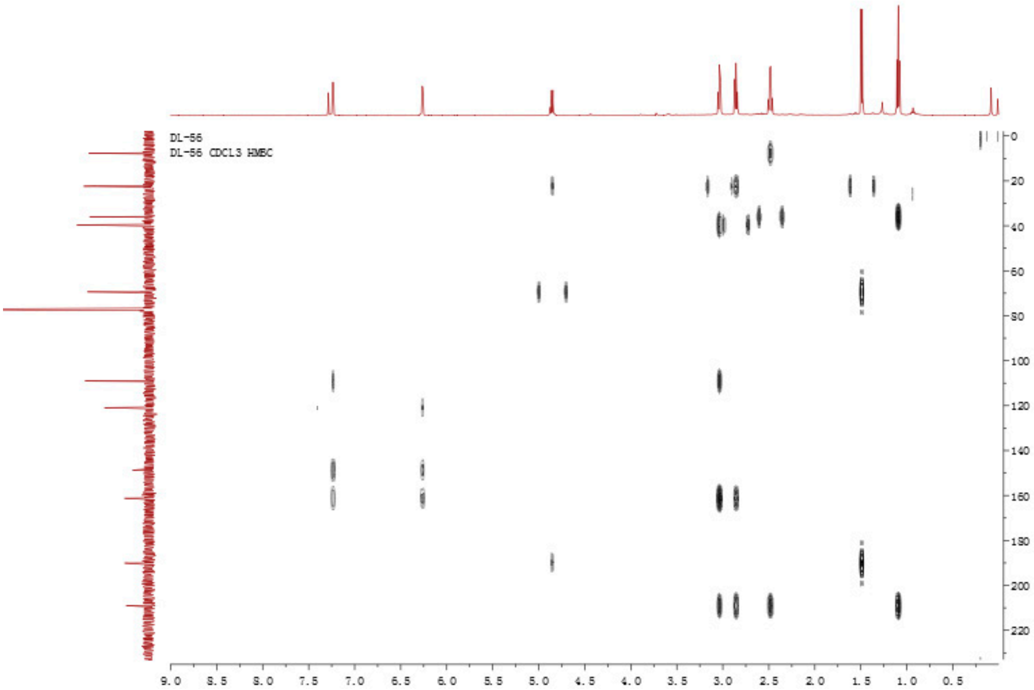


**Figure S12.** HMBC (500 MHz, $CDCl_3$) spectrum of 1-(5-(2-hydroxypropanoyl)-furan-2-yl)-pentan-3-one (**2**).

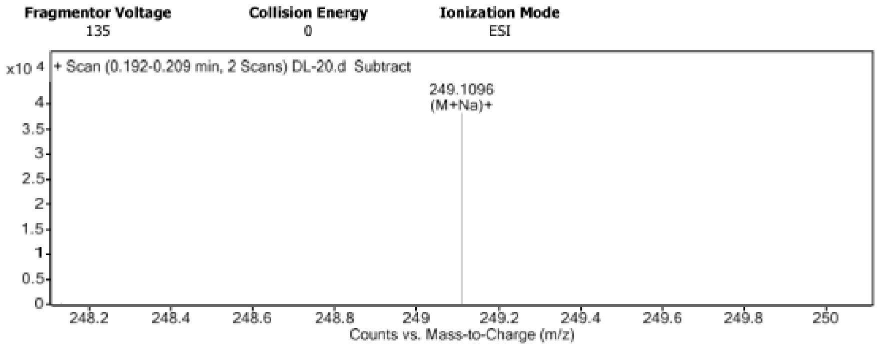


| Formula | CalculatedMass | CalculatedMz | Mz | Diff. (mDa) | Diff. (ppm) | DBE |
|---|---|---|---|---|---|---|
| C12 H18 O4 | 226.1205 | 249.1097 | 249.1096 | -0.1 | -0.4 | 4.0000 |

**Figure S13.** HRESIMS spectrum of 2-hydroxy-1-(5-(1-hydroxypentyl)-furan-2-yl)-propan-1-one (**3**).

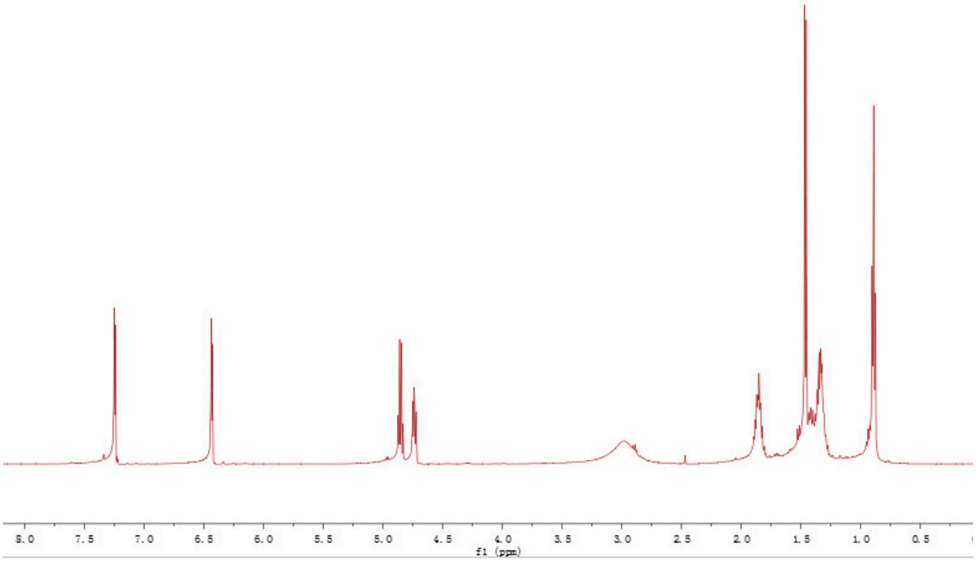


**Figure S14.** $^1$H-NMR (500 MHz, $CDCl_3$) spectrum of 2-hydroxy-1-(5-(1-hydroxypentyl)-furan-2-yl)-propan-1-one (**3**).

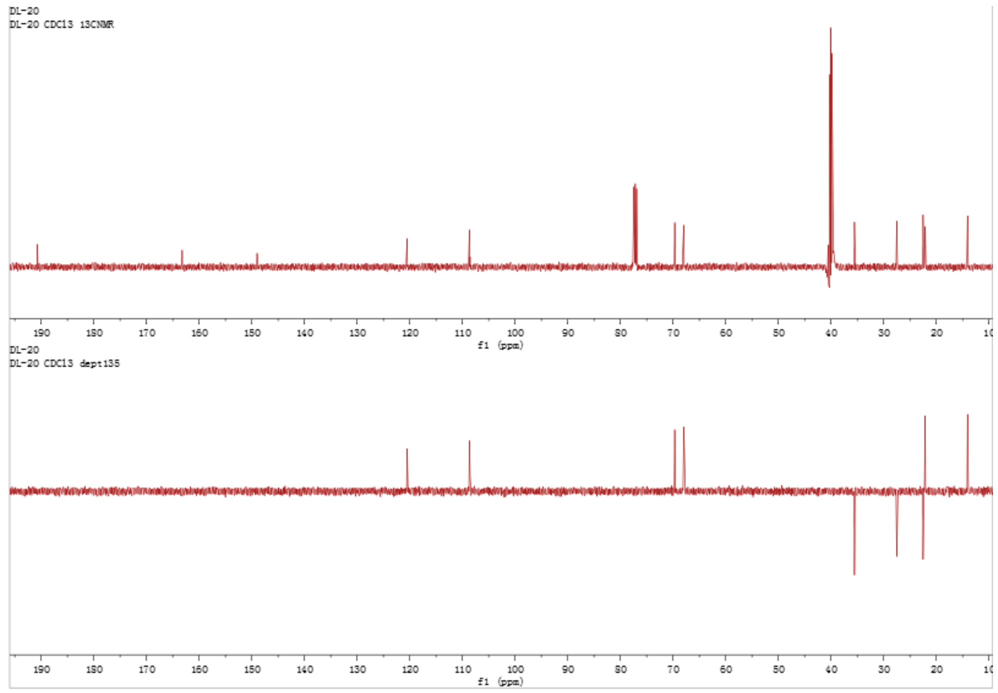


**Figure S15.** $^{13}$C-NMR (125 MHz, $CDCl_3$) spectrum of 2-hydroxy-1-(5-(1-hydroxypentyl)-furan-2-yl)-propan-1-one (**3**).

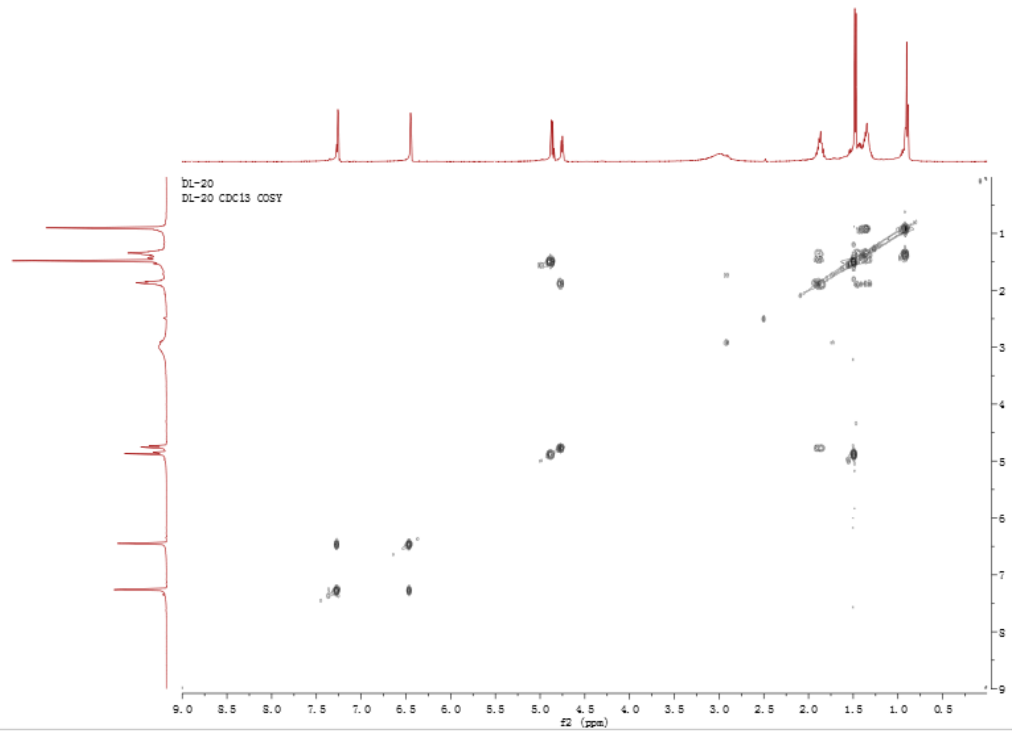


**Figure S16.** $^{1}$H-$^{1}$H COSY (500 MHz, $CDCl_3$) spectrum of 2-hydroxy-1-(5-(1-hydroxypentyl)-furan-2-yl)-propan-1-one (**3**).

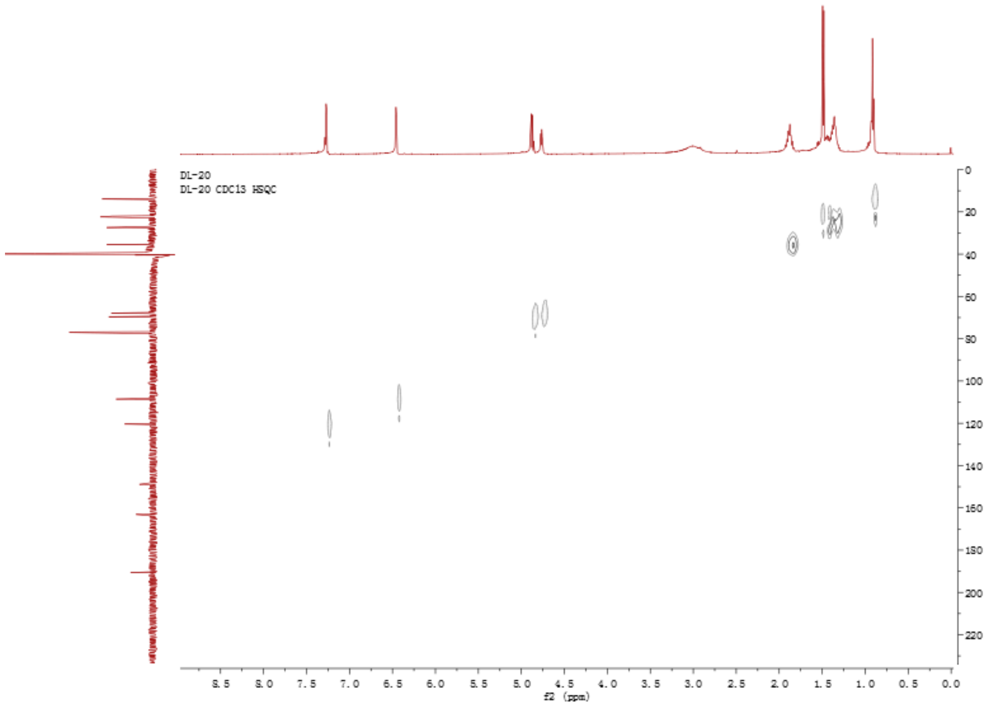


**Figure S17.** HSQC (500 MHz, $CDCl_3$) spectrum of 2-hydroxy-1-(5-(1-hydroxypentyl)-furan-2-yl)-propan-1-one (**3**).

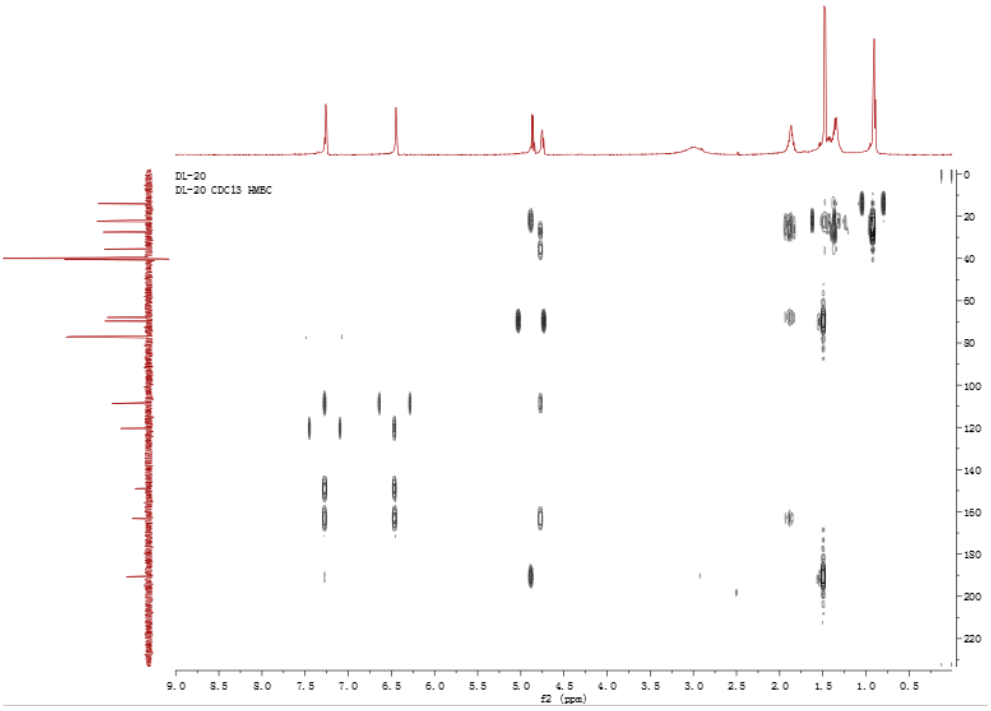


**Figure S18.** HMBC (500 MHz, $CDCl_3$) spectrum of 2-hydroxy-1-(5-(1-hydroxypentyl)-furan-2-yl)-propan-1-one (**3**).

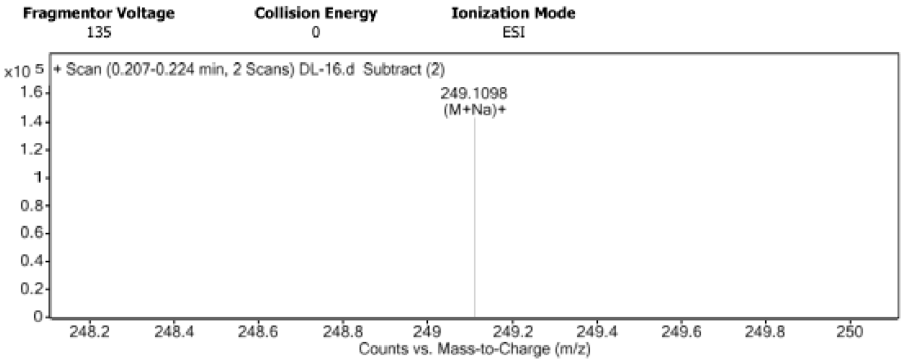


| Formula | CalculatedMass | CalculatedMz | Mz | Diff. (mDa) | Diff. (ppm) | DBE |
|---|---|---|---|---|---|---|
| C12 H18 O4 | 226.1205 | 249.1097 | 249.1098 | -0.1 | -0.3 | 4.0000 |

**Figure S19.** HRESIMS spectrum of 1-(5-(1,2-dihydroxypropyl)-furan-2-yl)-pentan-1-one (**4**).

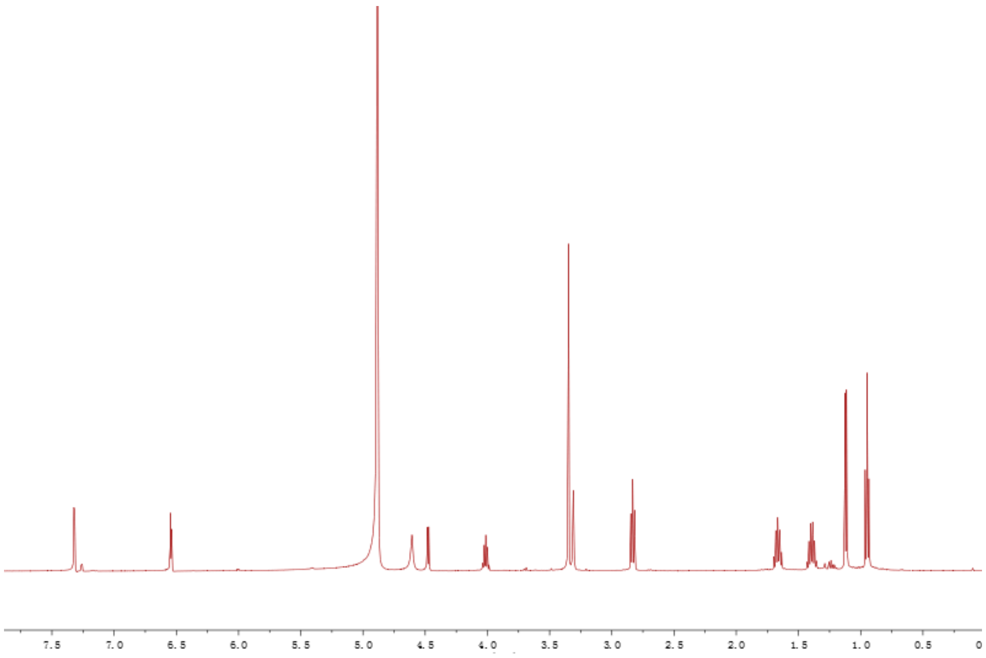


**Figure S20.** $^{1}$H-NMR (500 MHz, $CD_3OD$) spectrum of 1-(5-(1,2-dihydroxypropyl)-furan-2-yl)-pentan-1-one (**4**).

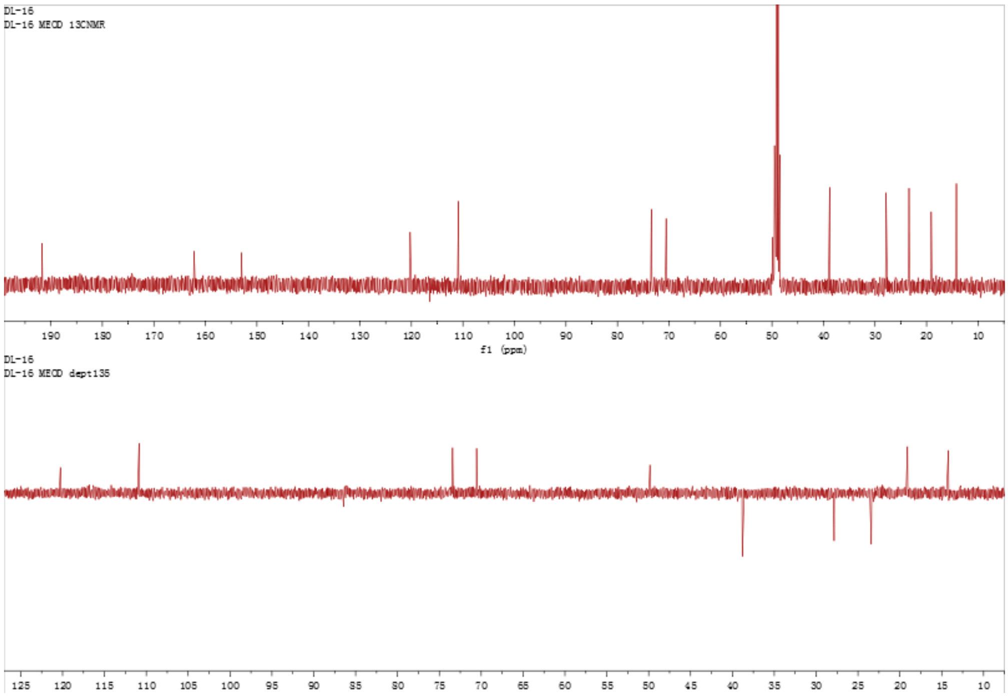


**Figure S21.** $^{13}C$-NMR (125 MHz, $CD_3OD$) spectrum of 1-(5-(1,2-dihydroxypropyl)-furan-2-yl)-pentan-1-one (**4**).

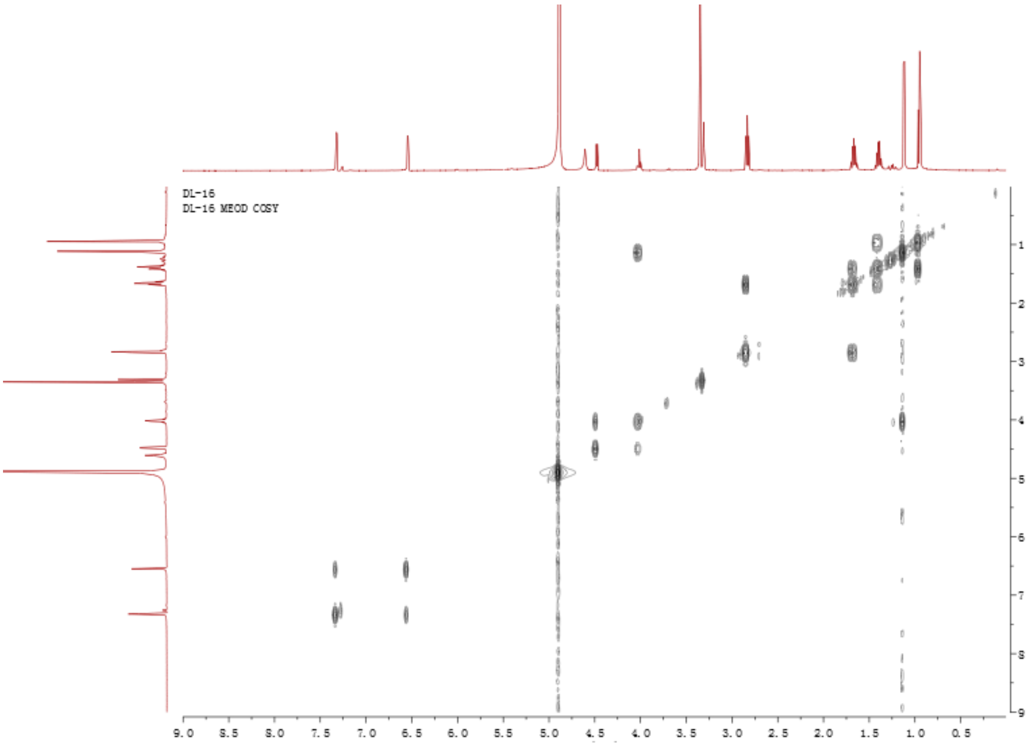


**Figure S22.** $^1H$-$^1H$ COSY (500 MHz, $CD_3OD$) spectrum of 1-(5-(1,2-dihydroxypropyl)-furan-2-yl)-pentan-1-one (**4**).

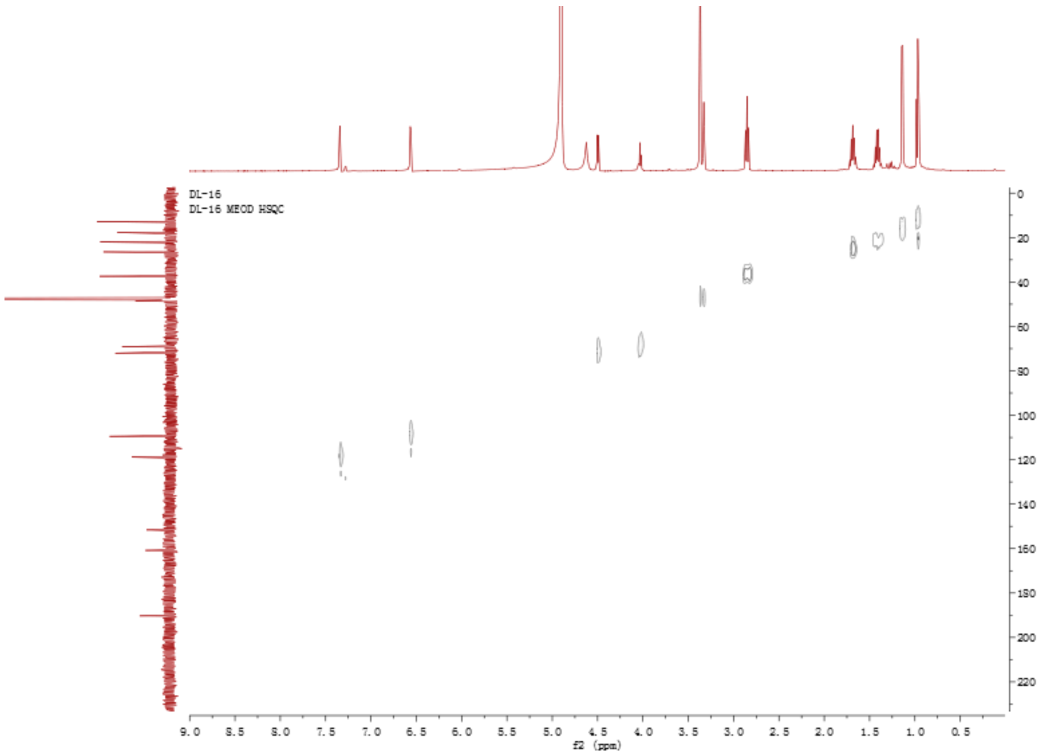


**Figure S23.** HSQC (500 MHz, $CD_3OD$) spectrum of 1-(5-(1,2-dihydroxypropyl)-furan-2-yl)-pentan-1-one (**4**).

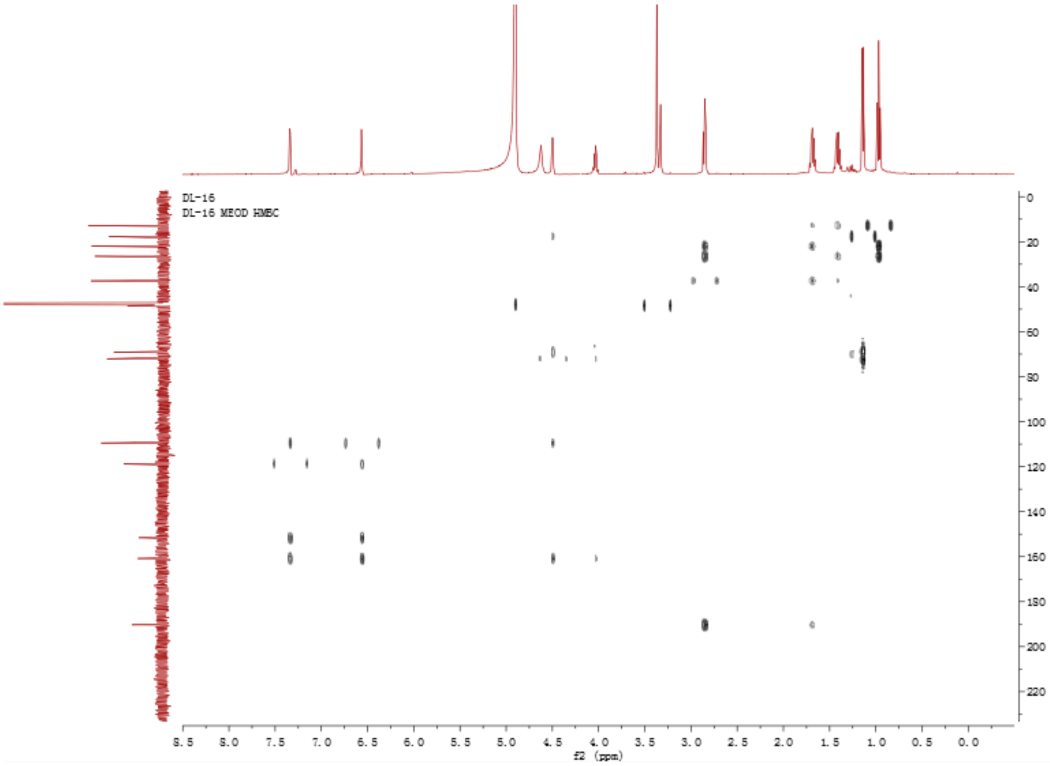


**Figure S24.** HMBC (500 MHz, $CD_3OD$) spectrum of 1-(5-(1,2-dihydroxypropyl)-furan-2-yl)-pentan-1-one (**4**).

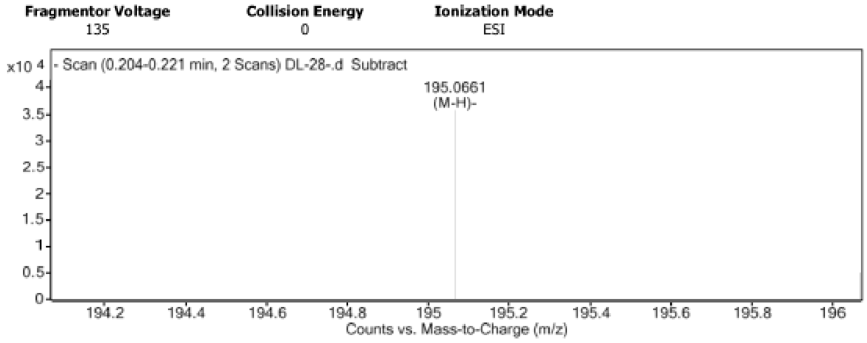


| Formula | CalculatedMass | CalculatedMz | Mz | Diff. (mDa) | Diff. (ppm) | DBE |
|---|---|---|---|---|---|---|
| C10 H12 O4 | 196.0736 | 195.0663 | 195.0661 | 0.0 | -0.1 | 5.0000 |

**Figure S25.** HRESIMS spectrum of 5-(1-hydroxypent-4-en-1-yl)-furan-2-carboxylic acid (**5**).

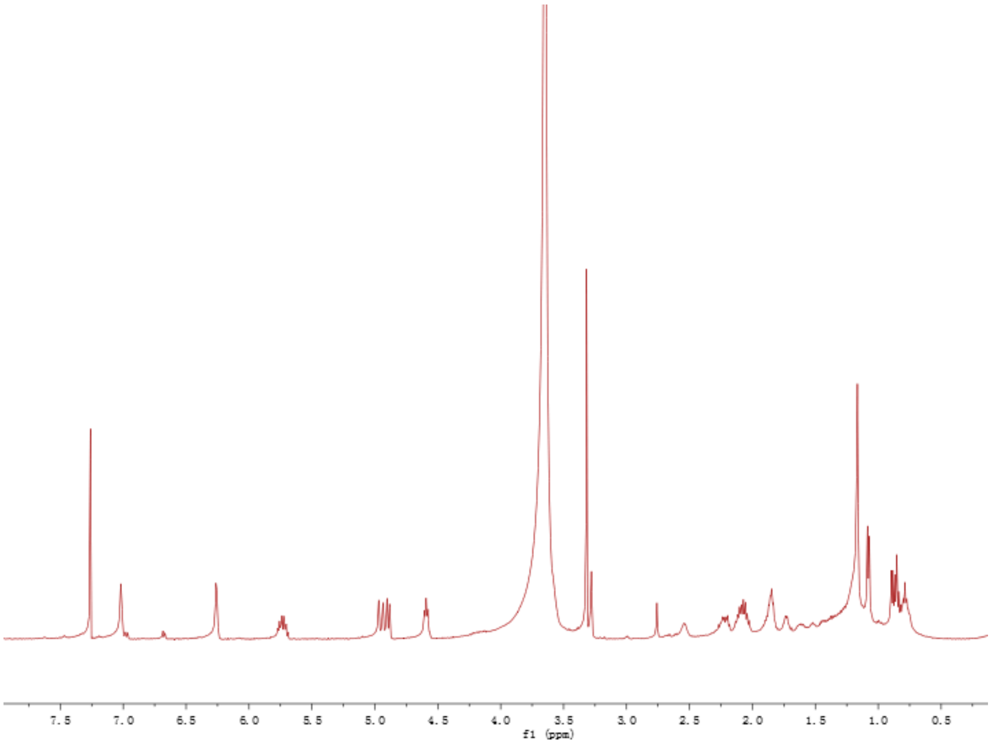


**Figure S26.** $^1$H-NMR (500 MHz, $CDCl_3$) spectrum of 5-(1-hydroxypent-4-en-1-yl)-furan-2-carboxylic acid (**5**).

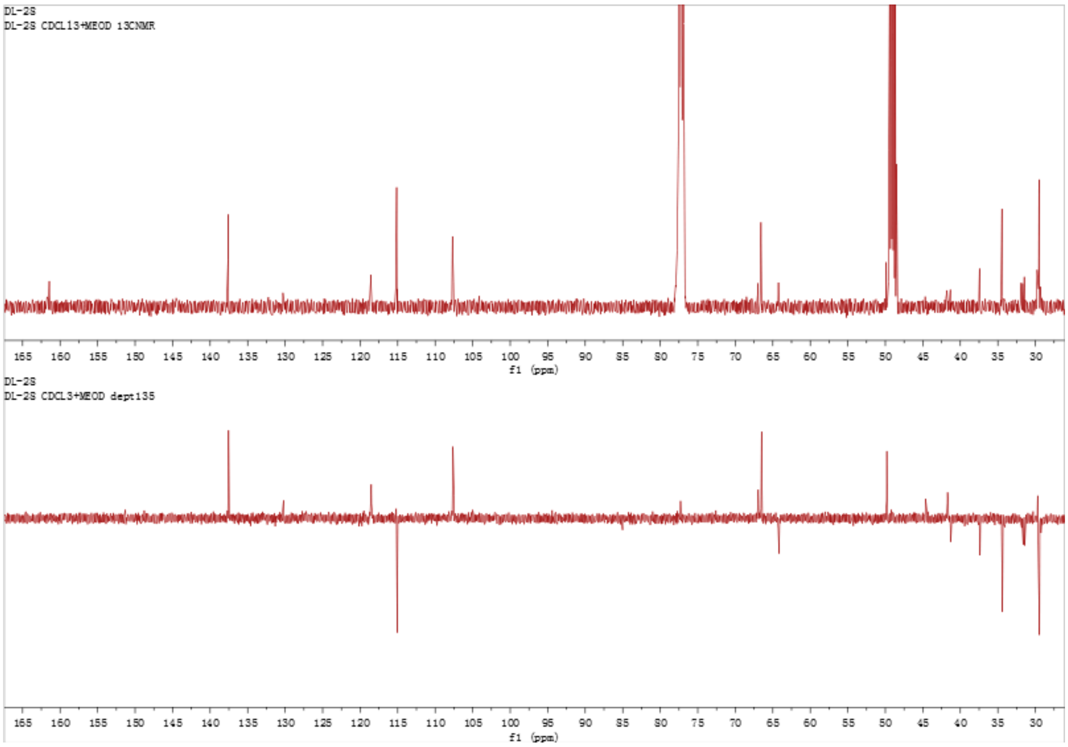


**Figure S27.** $^{13}$C-NMR (125 MHz, $CDCl_3$) spectrum of 5-(1-hydroxypent-4-en-1-yl)-furan-2-carboxylic acid (**5**).

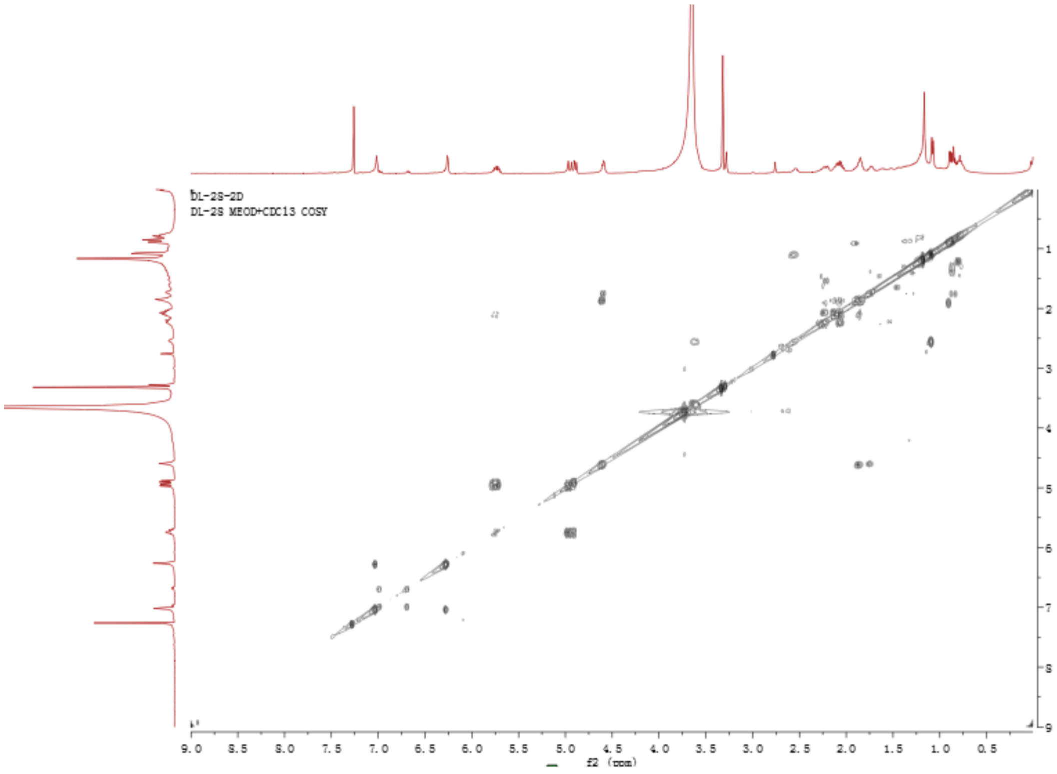


**Figure S28.** $^1$H-$^1$H COSY (500 MHz, $CDCl_3$) spectrum of 5-(1-hydroxypent-4-en-1-yl)-furan-2-carboxylic acid (**5**).

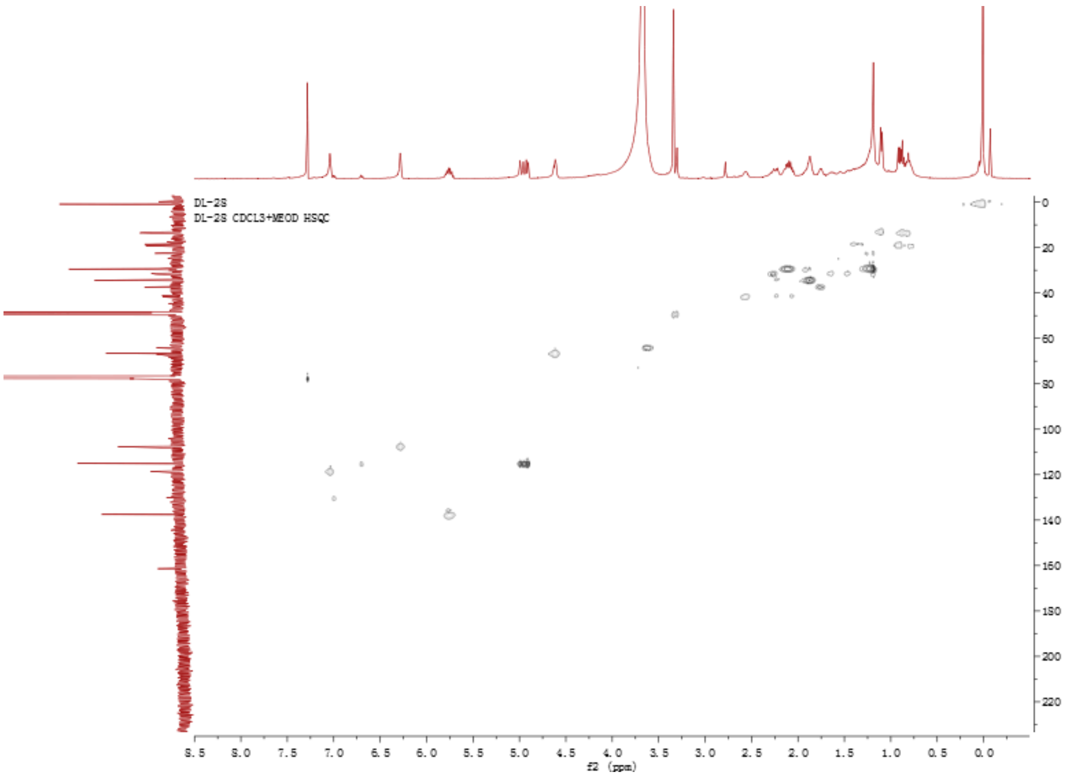

**Figure S29.** HSQC (500 MHz, $CDCl_3$) spectrum of 5-(1-hydroxypent-4-en-1-yl)-furan-2-carboxylic acid (**5**).

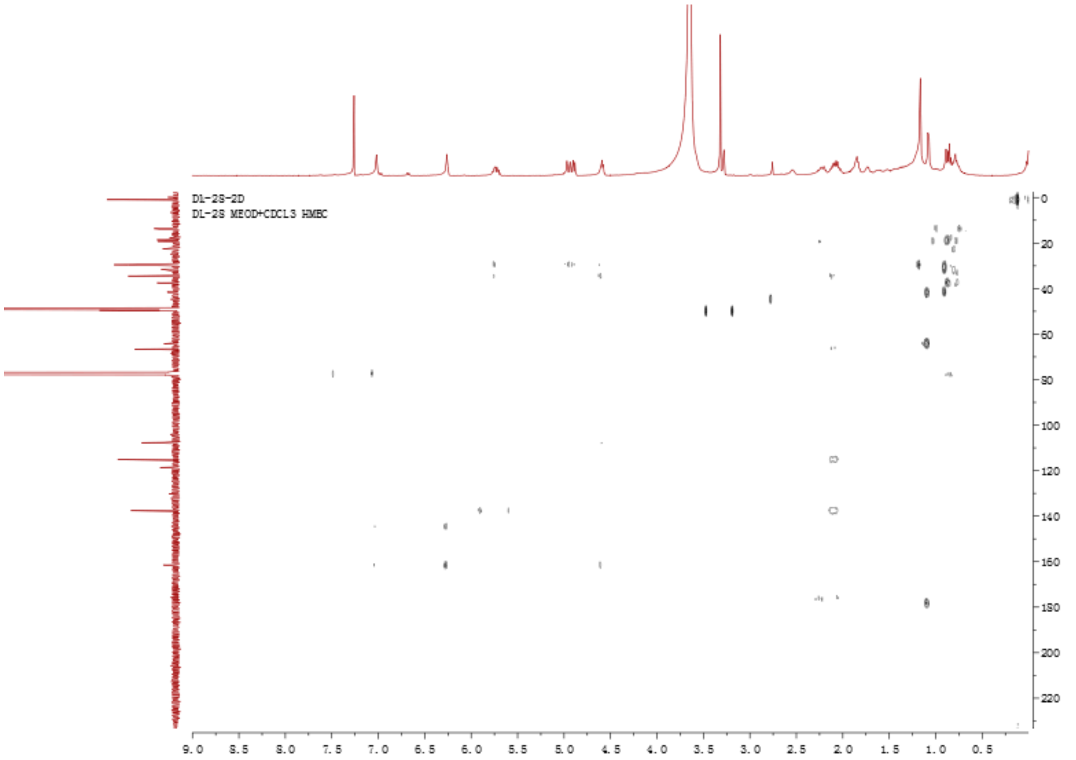

**Figure S30.** HMBC (500 MHz, $CDCl_3$) spectrum of 5-(1-hydroxypent-4-en-1-yl)-furan-2-carboxylic acid (**5**).

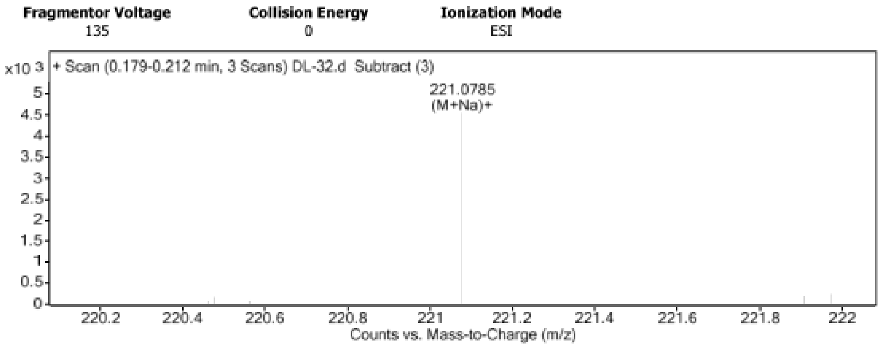


| Formula | CalculatedMass | CalculatedMz | Mz | Diff. (mDa) | Diff. (ppm) | DBE |
|---|---|---|---|---|---|---|
| C10 H14 O4 | 198.0892 | 221.0784 | 221.0785 | 0.0 | 0.0 | 4.0000 |

**Figure S31.** HRESIMS spectrum of 5-(3-hydroxypentyl)-furan-2-carboxylic acid (**6**).

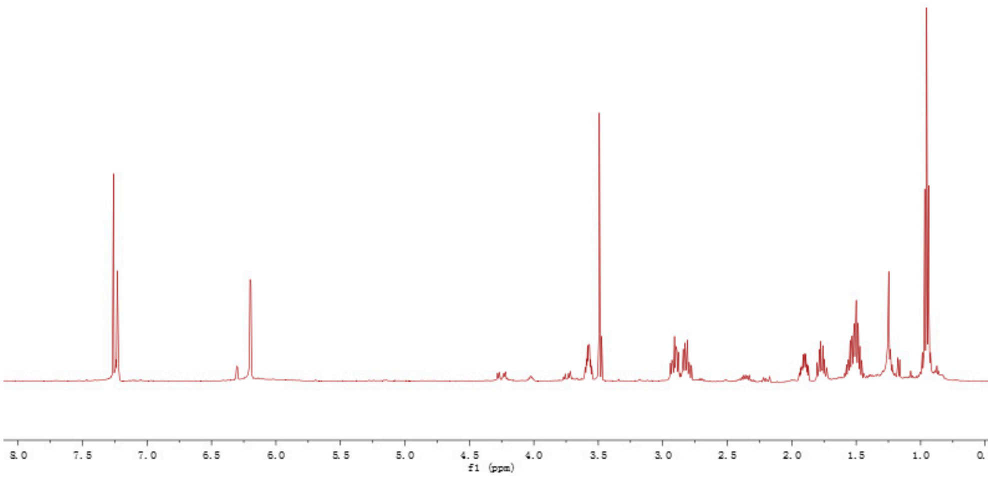


**Figure S32.** $^{1}$H-NMR (500 MHz, $CDCl_3$) spectrum of 5-(3-hydroxypentyl)-furan-2-carboxylic acid (**6**).

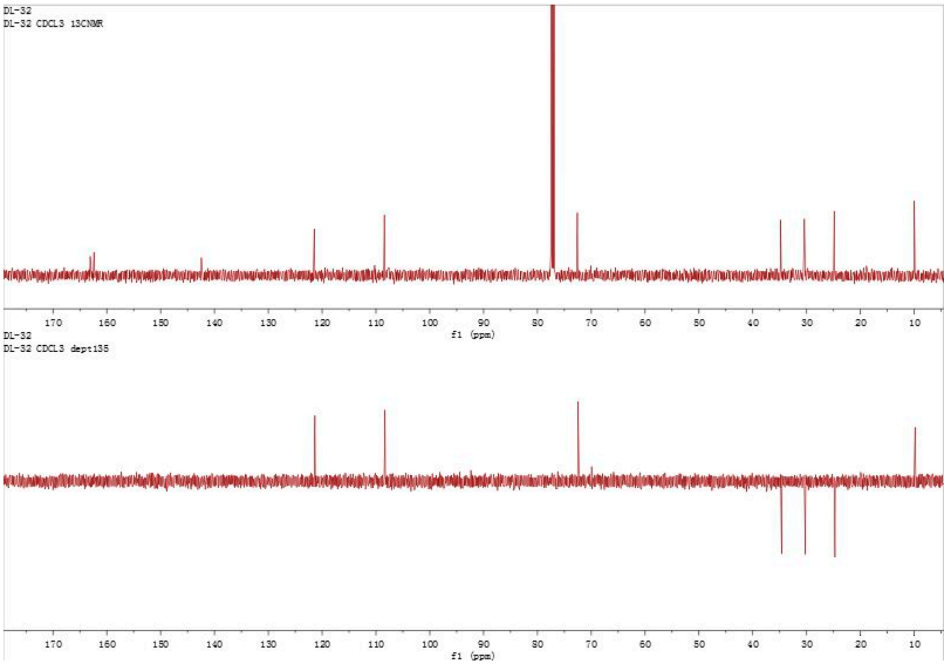


**Figure S33.** $^{13}$C-NMR (125 MHz, $CDCl_3$) spectrum of 5-(3-hydroxypentyl)-furan-2-carboxylic acid (**6**).

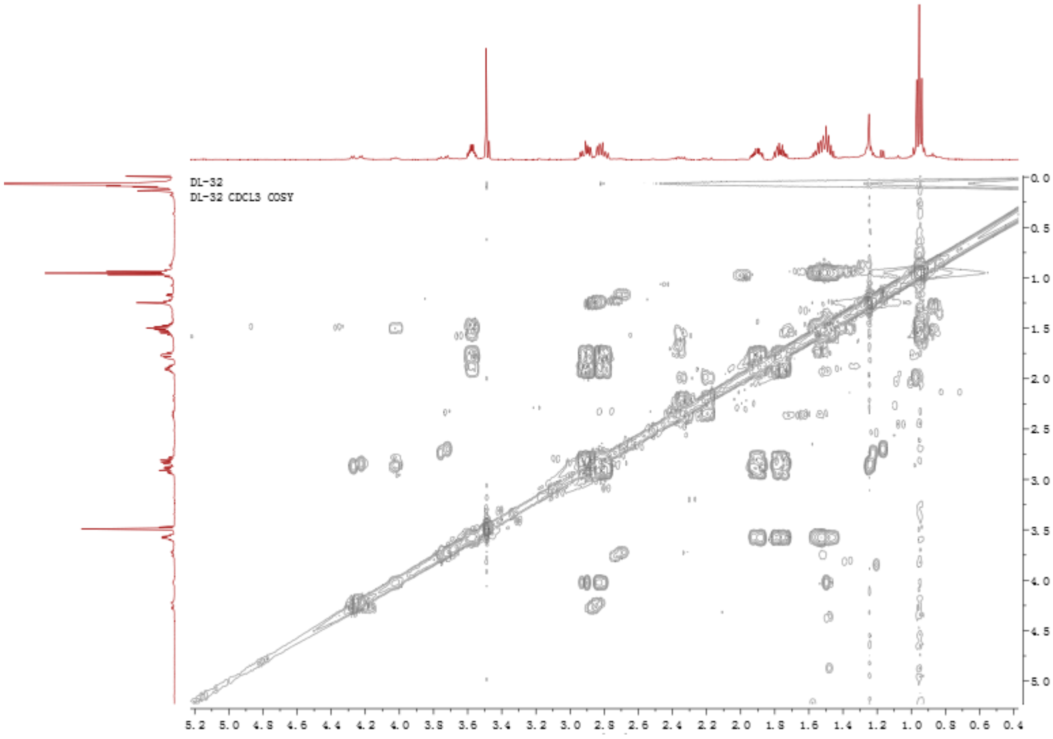


**Figure S34.** $^{1}$H-$^{1}$H COSY (500 MHz, $CDCl_3$) spectrum of 5-(3-hydroxypentyl)-furan-2-carboxylic acid (**6**).

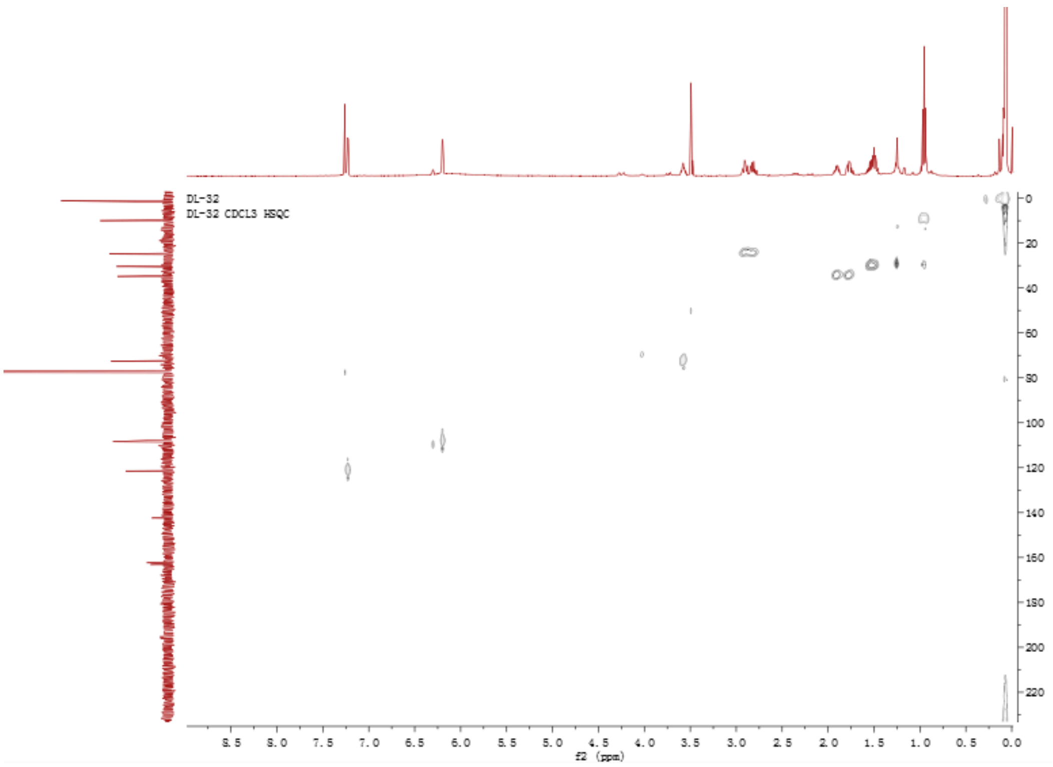


**Figure S35.** HSQC (500 MHz, $CDCl_3$) spectrum of 5-(3-hydroxypentyl)-furan-2-carboxylic acid (**6**).

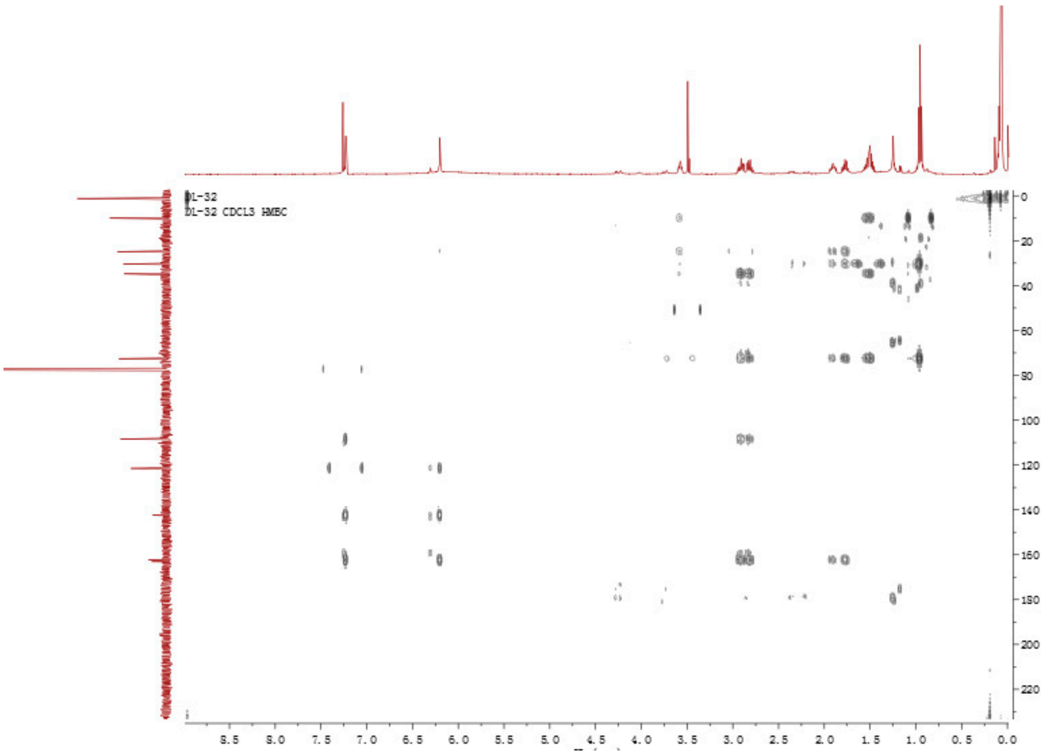


**Figure S36.** HMBC (500 MHz, $CDCl_3$) spectrum of 5-(3-hydroxypentyl)-furan-2-carboxylic acid (**6**).
